# 53BP1 Protects against CtIP-Dependent Capture of Ectopic Chromosomal Sequences at the Junction of Distant Double-Strand Breaks

**DOI:** 10.1371/journal.pgen.1006230

**Published:** 2016-10-31

**Authors:** Josée Guirouilh-Barbat, Camille Gelot, Anyong Xie, Elodie Dardillac, Ralph Scully, Bernard S. Lopez

**Affiliations:** 1 CNRS UMR 8200, Institut de Cancérologie Gustave Roussy, Université Paris Sud, Equipe labélisée “LIGUE 2014”, Rue Edouard Vaillant; 2 Department of Medicine, Harvard Medical School, Beth Israel Deaconess Medical Center, Boston; The University of North Carolina at Chapel Hill, UNITED STATES

## Abstract

DNA double-strand breaks (DSB) are very harmful lesions that can generate genome rearrangements. In this study, we used intrachromosomal reporters to compare both the efficiency and accuracy of end-joining occurring with close (34 bp apart) *vs*. distant DSBs (3200 bp apart) in human fibroblasts. We showed that a few kb between two intrachromosomal I-SceI-induced DSBs are sufficient to foster deletions and capture/insertions at the junction scar. Captured sequences are mostly coupled to deletions and can be partial duplications of the reporter (i.e., sequences adjacent to the DSB) or insertions of ectopic chromosomal sequences (ECS). Interestingly, silencing 53BP1 stimulates capture/insertions with distant but not with close double-strand ends (DSEs), although deletions were stimulated in both case. This shows that 53BP1 protects both close and distant DSEs from degradation and that the association of unprotection with distance between DSEs favors ECS capture. Reciprocally, silencing CtIP lessens ECS capture both in control and 53BP1-depleted cells. We propose that close ends are immediately/rapidly tethered and ligated, whereas distant ends first require synapsis of the distant DSEs prior to ligation. This "spatio-temporal" gap gives time and space for CtIP to initiate DNA resection, suggesting an involvement of single-stranded DNA tails for ECS capture. We therefore speculate that the resulting single-stranded DNA copies ECS through microhomology-mediated template switching.

## Author Summary

A DNA double-strand break is a very toxic lesion that can be repaired by rejoining DNA ends. This repair process can have deleterious consequences on the genome by joining DNA ends that were not originally fused (translocations) or modifying the DNA sequence with deletions or insertions. Here, we show that rejoining distant ends (only a few kb apart) favors error-prone repair characterized by deletion of the original sequences and also favors insertions of ectopic chromosomal sequences. These insertions are coupled to error-prone repair, i.e., deletion of the original sequence, initiated by the nuclease CtIP. Interestingly, favoring deletions by removal of the protection factor 53BP1 is not sufficient to efficiently promote insertions of ectopic sequences when the DNA ends are close. Therefore, the association of both unprotection and distance between DNA ends favors insertion of ectopic chromosomal sequences. The requirement of CtIP that generates single-strand DNA suggests that the generation of single-strand DNA favors insertions of ectopic sequences by microhomology-mediated template switching.

## Introduction

DNA double-strand breaks (DSBs) are highly toxic lesion that can cause profound genome rearrangements and/or cell death. Faithful DSB repair is vital for cell survival and the maintenance of genome stability, but it should also allow for genetic diversity in essential physiological processes such as, for instance, the establishment of the immune repertoire. Thus, DSB repair should be tightly controlled. There are two levels of genetic modification through DSB repair: 1- the rearrangement/joining of distant DNA sequences and 2- mutagenesis at the sealed junction. In this latter case, deletions, DNA capture or complex events, associating different processes can alter the structure of the repair junction.

DSBs are repaired by two general processes: the first uses an intact homologous sequence and is referred to as homologous recombination (HR), and the second process joins the two DNA double-strand ends (DSE) in a sequence homology-independent manner [1]. In mammalian cells, the end-joining (EJ) of DSEs is a prominent DSB repair pathway. Canonical non-homologous end-joining (C-NHEJ), which is KU-Ligase 4 dependent, is able to join DSEs in a conservative way at the repair junction, although it is adaptable to imperfectly cohesive ends [2–4]. More recently, an alternative end-joining (A-EJ) pathway has been described that does not require sequence homology, but is initiated by CtIP-dependent single-strand DNA resection. Therefore, A-EJ is highly mutagenic at the repair junction, typically generating deletions resulting from the initial resection and frequently using microhomologies distant from the DSB to join the resected DNA ends [2–10].

Several mechanisms generate DSBs: DNA-damaging agents, such as ionizing radiation or reactive oxygen species, and nucleases generate DSBs with two proximal DSEs. Prolonged replication stress also generates DSBs [11]. Importantly, the arrest of replication forks generates single-ended DSEs [12,13], and the joining of such structures, which are distant, inevitably generates rearrangements. Importantly, we have recently reported that the cohesin complex prevents the joining of distant double-strand ends but not of close ends, specifically in the S phase [14]. Alternatively, single-ended DSEs can initiate DNA copy through template-switching. Indeed, a model of genetic rearrangements accounting for copy number variation upon replication stress, which is initiated by microhomology annealing, has been proposed: MMBIR/FoSTeS (microhomology-mediated break-induced replication/ Fork Stalling and Template Switching) [15–17]. In yeast, chromosome rearrangements occurring via template switching between diverged repeated sequences have also been described [18].

On one DSB with two proximal DSEs, it has been proposed that C-NHEJ components tether the two ends, allowing their immediate ligation [19–22]. Remarkably, C-NHEJ-defective cells exhibit strong chromosome instability, underlining the fact that C-NHEJ is essential for the maintenance of genome stability. Consistently, C-NHEJ protects against the mobility of DNA ends, thus preventing unscheduled rearrangements [23–25]. Conversely, A-EJ is involved in chromosome translocation in mouse, drosophila and yeast cells [26–28]. However, the mechanisms leading to genome rearrangements appear to be more complex because C-NHEJ has also been shown to be involved in genome rearrangement events such as capture of excised chromosomal sequences and translocation, in the mammalian genome [2,29]. Moreover, analysis of the junctions repaired by EJ reveals the occurrence of complex events in addition to the direct joining of two DSEs. Indeed, these events frequently associate deletions with capture of DNA sequences. Moreover, while the genetic control of the end-joining processes *per se* has been extensively studied [1], the mechanisms resulting in rearranged end-joining junctions are poorly documented.

Here, we address the question of the impact of the distance between two DSEs on both the efficiency and the accuracy of end joining. To analyze these processes at a precise molecular level in living human cells, and in the chromosomal context, we used several intrachromosomal substrates monitoring the end joining of DSBs targeted into the substrates by the meganuclease I-SceI. These substrates have been derived from previously extensively characterized, validated and discussed substrates monitoring end-joining [2–4,8,10,30–32]. We show that a distance of only a few kb between the two DSEs, which is short at the nucleus scale, is sufficient not only to significantly reduce joining efficiency but also to induce error-prone DSB repair associated with complexly rearranged end-joining junctions. Particularly, a distance between the DSEs favors the capture of chromosome sequences that can be partial duplications of the EJ reporter or ectopic chromosomal sequences (ECS). We show here that these captures are promoted by CtIP and counteracted by 53BP1, suggesting the involvement of single-strand resection at the initiation of such events. Therefore, according to these data, the junction patterns analyzed here, the MMBIR/Fostes model [15–17], and analysis of chromosome rearrangement in yeast [18], we speculate that the chromosomal captures at the end-joining junctions of two distant DSEs also result from micro-homology-mediated template switching. These complex events only arise with distant DSEs, thereby indicating a requirement for a "spatio-temporal-gap" that allows the coupling of the resection with chromosomal insertions. These data reveal mechanisms resulting in DNA capture at the joining of two distant DSBs, underlining the complex possibilities for DNA end processing to alter the accuracy of DSB repair. Importantly, even a distance of a few kb between two DSBs is sufficient to induce such complex processing, adding an additional level of risk for genome integrity.

## Results

### A few kb between two DSBs are sufficient to affect EJ efficiency

**Fig 1:**
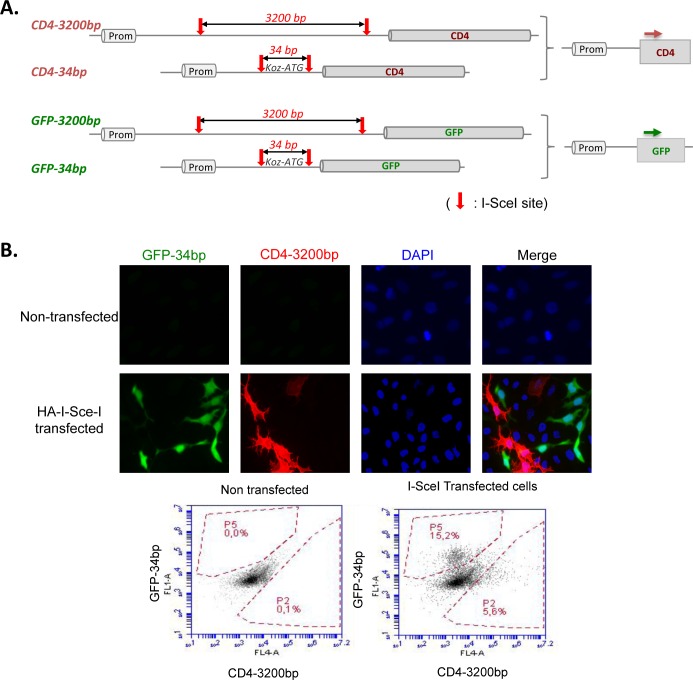
End-joining substrates. **A.** CD4-3200bp and GFP-34bp have previously been validated [10,34–36,48] and discussed in [4]. To analyze DSB repair with comparable substrate backbones, we constructed hybrid substrates, CD4-34bp and GFP-3200bp interchanging the internal sequences flanked by the two I-SceI sites (red arrows). After I-SceI cleavage, the end-joining leads to expression of the reporters (see details in S1 Supplementary information). **B.** Example of GFP and CD4 monitoring by fluorescence microscopy (upper panel) or flow cytometry (lower panel), in a given cell line (GCS5).

We designed several intrachromosomal reporter substrates monitoring non-homologous EJ, between which the key difference was the distance between the two DSEs (I-SceI sites), 34 bp *versus* 3200 bp (Fig 1A and S1 supplementary information). A 34-bp-gap should allow for more direct or rapid tethering and ligation of the two DSEs. In contrast, a 3200-bp-gap absolutely requires a synapsis step to bring together the two DSEs prior to ligation. End-joining events were monitored by the expression of GFP or CD4 reporters (Fig 1B).

**Fig 2:**
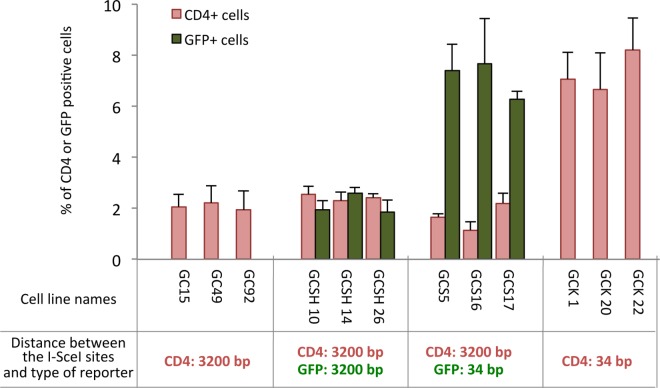
The distance between DSB affects the efficiency of end joining. **A.** Frequency of end joining in cell lines bearing one or two substrates: 12 independent clones contained one or two of the substrates. Values represent the average ± SEM of at least 5 independent experiments.

We established several independent clones bearing one or two substrates in SV40-transformed human fibroblasts (Fig 2). Note that, for a given type of substrate, the frequency of I-SceI–induced EJ did not significantly vary between different clones with the same reporter type, suggesting the absence of position effect (Fig 2). Remarkably, the efficiency of EJ was consistently 3.5-fold higher in reporters containing a 34-bp-gap than in those containing a 3200-bp-gap (Fig 2). This shows that even a few kilobases of separation between DSEs, which is short at the genome-scale level, reduce EJ efficiency; this effect is therefore not restricted to large-scale genomic rearrangements [33].

### A few kb between two DSBs affect the accuracy of EJ

In previous studies, we defined two classes of EJ repair events: conservative EJ (C-NHEJ), which is KU/XRCC4-dependant and uses the annealing of at least one of the 3’ protruding nucleotides (3’Pnt) generated by I-SceI cleavage, which are eventually associated with insertions at the repair junctions; and non-conservative alternative EJ (A-EJ), which is KU/XRCC4-independant and deletes at least all four 3’-Pnt (see S1 supplementary information and [4,10,34–36]). Again, deletions in A-EJ can be associated with insertions at the repair junction. DSEs separated by 34 bp produced a higher proportion of conservative rejoining events (64% conservative repair: 57% HiFi (High Fidelity events, i.e. error-free) + 7% insertions in GCK20 cells) than in each of the two cell lines (GC92 and GC49) with DSEs separated by 3200 bp (40% conservative repair: 36% HiFi+4% insertions in GC92 cells, and 33% conservative repair: 23% HiFi+ 10% insertions in GC49 cells) (GC92 *vs*. GCK20,p = 0.007; GC49 *vs*. GCK20, p = 0.005 by t-test; Table 1 and S2 Supplementary information), suggesting that distance between DSEs fosters error-prone repair, likely by A-EJ.

**Table 1:** Accuracy of End Joining on distal or close DSEs.

	DISTANT DSEs CD4-3200bp	DISTANT DSEs CD4-3200bp	CLOSE DSEs CD4-34bp
	GC92 cells	GC49 cells	GCK20 cells
Number of sequences	190	80	135
HiFi (High Fidelity)	36%	23%	57%
	(69/190)	(18/80)	(77/135)
Insertion ≥1bp	4%	10%	7%
	(7/190)	(8/80)	(9/135)
Deletion ≥1bp	46%	47%	26%
	(87/190)	(38/80)	(35/135)
Deletion with insertion	14%	20%	10%
	(27/190)	(16/80)	(14/135)

HiFi (High Fidelity) is the direct ligation of 1 to 4 of the 3’protruding nucleotides generated by the I-SceI cleavage. Insertions are all reported even as small as 1bp. Deletions include deletions of nucleotides located on the double-stranded side of the DSE. Values are calculated from the total of at least 3 independent experiments and sequencing of 80 to 190 junction sequences. (T-test of the use of 3’Pnt (HiFi+ insertions): GC92 *vs*. GCK20: p = 0.0034; GC49 *vs*. GCK20: p = 0.0068).

**Fig 3:**
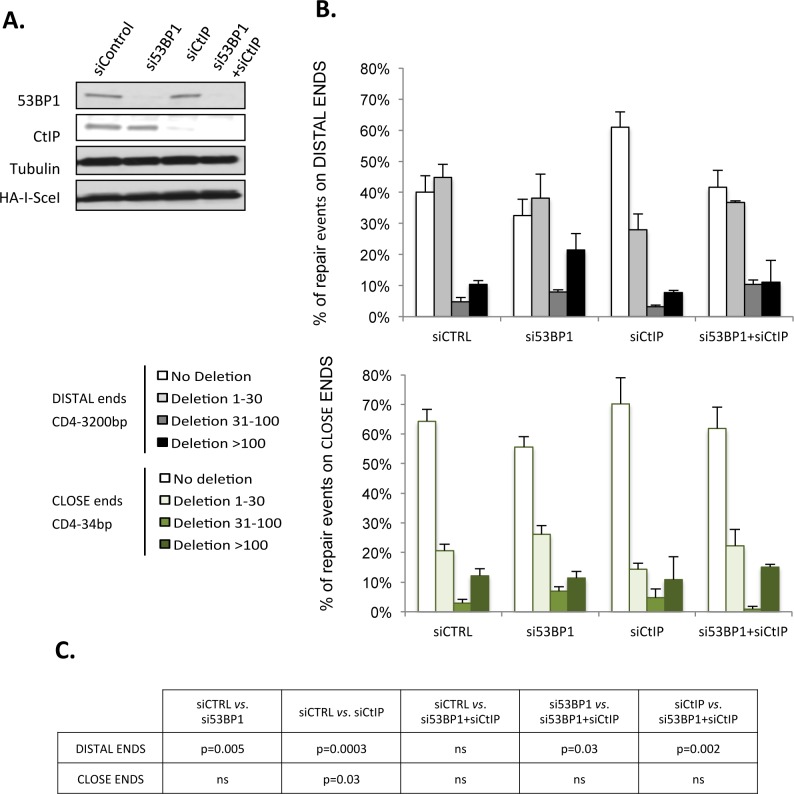
53BP1 protects against non-conservative end-joining on both distant and close ends but counteracts CtIP mediated resection only on distant ends. **A.** Protein expression in cells transfected with control siRNA and/ or 53BP1 and/or CtIP siRNAs. **B.** Distribution of the size of deletion at the repair sites of distant ends (upper panel, GC92 cells) *vs*. close ends (lower panel, GCK20 cells) in cells transfected with 53BP1 siRNA and/or CtIP siRNA. Values represent the mean +/- SEM of 2 to 5 experiments and analysis of 78 to 190 sequences. **C.** The p values (Mann and Whitney test) from Fig 3B.

We previously showed that CtIP promotes non-conservative rejoining of DSEs separated by 3200 bp and that 53BP1 antagonizes CtIP in this process [36]. Here, we reproduced these results (Table 2), and in addition, we showed that silencing CtIP (Fig 3A) also increases conservative events (HiFi) with DSEs separated by 34 bp (Table 2). Silencing 53BP1 (Fig 3A), which protects against CtIP-induced resection [37], increased the percentage of non-conservative events with distant ends (Table 2) and significantly increased the size of deletions (Fig 3B).

**Table 2:** Accuracy of End Joining on distant or close DSEs, upon 53BP1 and/or CtIP depletion.

	DISTANT DSEs				DISTANT DSEs		CLOSE DSEs			
	CD4-3200bp				CD4-3200bp		CD4-34 bp			
	GC92 cells				GC49 cells		GCK20 cells			
	siControl	si53BP1	siCtIP	si53BP1	siControl	si53BP1	siControl	si53BP1	siCtIP	si53BP1
				+siCtIP						+siCtIP
Number of sequences	190	163	165	95	80	123	135	120	86	78
HiFi	**36%**	**29%**	**57%**	**42%**	**23%**	**16%**	**57%**	**52%**	**69%**	**58%**
	(69/190)	(48/163)	(94/165)	(40/95)	(18/80)	(20/123)	(77/135)	(63/120)	(59/86)	(45/79)
Insertion ≥1bp	4%	3%	2%	0%	10%	2%	7%	4%	5%	1%
	(7/190)	(5/163)	(4/165)	(0/95)	(8/80)	(3/123)	(9/135)	(5/120)	(4/86)	(1/78)
Deletion ≥1bp	46%	45%	32%	44%	47%	58%	26%	31%	17%	29%
	(87/190)	(73/163)	(52/165)	(42/95)	(38/80)	(71/123)	(35/135)	(37/120)	(15/86)	(23/79)
Deletion with insertion	14%	23%	9%	14%	20%	24%	10%	13%	9%	12%
	(27/190)	(37/163)	(15/165)	(13/95)	(16/80)	(29/123)	(14/135)	(15/120)	(8/86)	(9/78)

Values are calculated from the total of 2 to 5 independent experiments and sequencing of 78 to 190 junction sequences.

With close ends, silencing 53BP1 (Fig 3A) also impaired conservative events (64%: 57% HiFi + 7% insertions in control cells *vs*. 56%: 52% HiFi + 4% insertions in 53BP1-depleted cells, Table 2); however, the size of deletions was not affected by 53BP1 depletion (Fig 3B). Therefore, 53BP1 is necessary to protect distant DSEs from extensive degradation. For close DSEs, unprotection by 53BP1 silencing is compensated by the tethering and rapid ligation of the two close ends, thus avoiding the attack of the DSE by nucleases and generation of long resections.

### A few kb between two DSBs favors the capture of ectopic chromosome sequences

**Fig 4:**
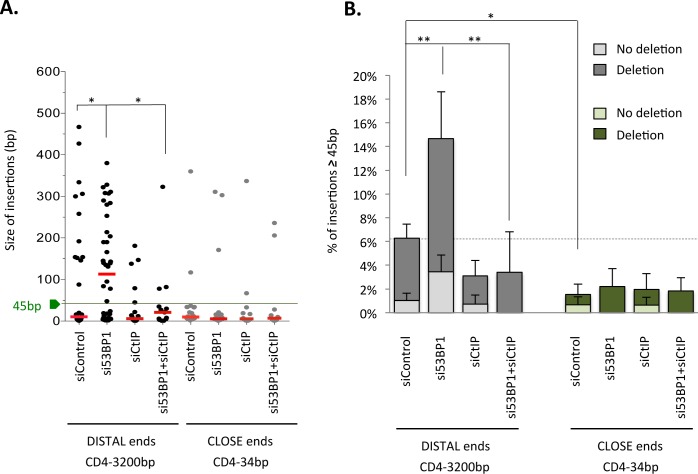
Long insertions are favored at the repair junction of unprotected distant DSEs. **A**. Impact of 53BP1 and/or CtIP depletion on the size of insertions at the repair sites of distant ends (GC92 cells) or close ends (GCK20 cells) in cells transfected with control siRNA and/ or 53BP1 and/or CtIP siRNAs. For each sample, each dot represents one insertion and the red line represents the median (*: p<0.03, Mann-Whitney test). The green line indicates the threshold of 45 bp that was chosen for sequence BLAST. **B.** Impact of 53BP1 and CtIP on the frequency of long insertions (≥45 bp). Histograms represent insertions coupled to a deletion event and insertions not coupled to a deletion event. Values represent the mean +/- SEM of at least 3 independent experiments and sequencing of 78 to 190 junction sequences. (*: p = 0.02; **: p<0.005, Mann-Whitney test).

Remarkably, rejoining 3200-bp-distant DSEs seems to significantly favor long insertions (≥ 45 bp and even >200 bp) compared to rejoining close 34-bp-distant ends (Fig 4A and 4B, and S2 supplementary information). Strikingly, silencing 53BP1 2.5-fold increased the frequency of these long insertions at the rejoining junction of 3200-bp-distant DSEs (Fig 4B, Tables 3 and 4, and S2 and S3 supplementary information). Interestingly, CtIP depletion decreased the frequency of long insertions in control cells but, more specifically, abolished the increased stimulation of long insertions resulting from 53BP1 depletion (Fig 4A and 4B and Table 3, S2 and S3 Supplementary information). In contrast, in the repair of 34-bp-separated DSEs, 53BP1 depletion had no impact on insertion size and frequency (Fig 4A and 4B and S3 Supplementary information). Collectively, the data show that deprotection of DSEs is not sufficient to efficiently promote insertions and that the distance between DSEs also matters.

**Table 3:** Origin of large insertions (>45 bp) monitored in the repair of distant DSEs (CD4-3200bp) upon 53BP1 and/or CtIP depletion, in the GC92 cell line.

			Origin of insertions >45 bp in the repair of DISTANT ends CD4-3200bp			
			GC92 cells			
	% of insertions >45 bp	% of insertions >45 bp coupled to a deletion >100 bp	Partial duplication of the EJ reporter	Ectopic chromosomal sequences (ECS)	Other	Insertions >45 bp bordered by unidentified nucleotides
siControl	**6.3%** (12/190)	5.3% (10/190)	2.1% (4/190)	4.2% (8/190)	-	83.3% (10/12)
si53BP1	**14.7%** (24/163)	9.8% (16/163)	6.1% (10/163)	7.4% (12/163)	1.2% (2/163)*	83.3% (20/24)
siCtIP	**3.0%** (5/165)	1.2% (2/165)	2.4% (4/165)	0.6% (1/165)	-	40.0% (2/5)
si53BP1+siCtIP	**3.1%** (3/96)	1.0% (1/96)	1.0% (1/96)	0.0% (0/96)	2.1% (2/96)**	66.7% (2/3)

Values are calculated from 2 to 5 independent experiments and sequencing of 96 to 190 junction sequences

* Bacterial DNA

** I-SceI expression vector DNA

**Table 4:** Origin of large insertions (>45 bp) monitored in the repair of distant DSEs (CD4-3200bp) upon 53BP1 depletion in a second cell line (GC49).

			Origin of insertions >45 bp in the repair of DISTANT ends CD4-3200bp			
			GC49 cells			
	% of insertions >45 bp	% of insertions >45 bp coupled to a deletion >100 bp	Partial duplication of the EJ reporter	Ectopic chromosomal sequences (ECS)	Other	Insertions >45 bp bordered by unidentified nucleotides
siControl	**2.4%** (2/81)	0.0% (0/81)	1.2% (1/81)	1.2% (1/81)	-	0.0% (0/2)
si53BP1	**6.5%** (8/123)	3.3% (4/123)	5.7% (7/123)	0.8% (1/123)	-	75.0% (6/8)

Values are calculated from 3 independent experiments and sequencing of 81 to 123 junction sequences

Sequencing of the insertional rejoining events of distant DSEs revealed that insertions could be classified into two main categories (Tables 3 and 4, S4 Supplementary information).

The first category entailed partial duplication of sequences adjacent (either in 5’ or in 3’) to the I-SceI cleavage site (31 sequences over 63 insertions ≥45 bp total). Among these events, four sequences implicated sequence homology at one border and copying of a part of the intervening sequence between the two I-SceI sites. Therefore, these rare events might be attributed to an HR-dependent process involving the sister chromatid. However, the vast majority (27/31 events of partial duplication of the EJ reporter) did not exhibit sequence homology at the borders nor copy of the intervening sequence immediately downstream from the I-SceI site. Therefore, for these latter cases, we exclude a process initiated by HR, and we propose that they occur through microhomologies-mediated unequal sister chromatid exchange, involving non-homologous sequences (see below). These events are similar to some of the translocation junctions observed in Ewing sarcoma [38]. The second category entailed capture of ectopic chromosomal sequences (ECS). In this latter category, there were also no sequence homologies between the donor and recipient DNA molecules observed, excluding the involvement of homologous recombination in the promotion of such events. Note that DNA capture has been described at translocation junctions involving two different chromosomes [39]. Importantly, for 3200-bp-separated DSEs, CtIP depletion abolished ECS capture (Table 3). Silencing 53BP1 stimulated the occurrence of long insertions (≥45 bp) in the two different cell lines we used here to monitor rejoining of distant ends, but the pattern differed between them. Indeed, silencing 53BP1 in the GC92 cell line stimulated both the partial duplication of the EJ reporter and ECS capture, whereas silencing 53BP1 only stimulated partial duplication of the reporter sequence in the GC49 cell line (Tables 3 and 4 and S4 Supplementary information). These differences may reflect a position effect and differences in chromatin conformation between the two different cell lines. However, the data conclude that ablation of 53BP1 leads to insertions at the seal junction of distant DSEs. For 34-bp-separated DSEs, the number of long insertions was very small in spite of the large number of repair events sequenced (only 2 and 3 insertions ≥45 bp among 135 and 120 sequences in control or 53BP1-depleted cells, respectively), but importantly, depletion of 53BP1 did not stimulate ECS capture, in contrast with 3200-bp-separated DSEs (Fig 4B, S5 Supplementary information). Intriguingly, captured ECS and partial duplications of the EJ reporter were frequently flanked by stretches of unidentified sequences (N-additions). Because these sequences are unidentified it is not possible to determine whether micro-homologies are involved; however, among the remaining events, which do not present unidentified sequences at the borders of the inserted sequence, approximately two-thirds exhibited micro-homologies (≥2 bp) at the junction borders (S4 Supplementary information).

## Discussion

Altogether, these data show that distance between DSEs not only decreases the efficiency of EJ but also its accuracy, favoring complex association/competition of different DNA end processing mechanisms (DNA degradation, synthesis, N-additions, and insertions) at the repair junction. Remarkably, although 3200 bp is a short distance at the whole genome scale, it is sufficient to generate such rearrangements. Determining the minimal distance requiring synapsis and the impact of longer distances on the efficiency and accuracy of joining represents an exciting challenge for future studies. Importantly, here, we identified genetic control of such events. Indeed, they are mainly CtIP-dependent and counteracted by 53BP1, suggesting a role for the single-stranded tails generated by resection of the DSEs. In yeast, increased mobility of DSBs has been associated with DNA end resection, thus favoring the search for homology during homologous recombination [40,41]. Moreover, in human cells, inhibition of MRE11 that initiates resection with CtIP in A-EJ [7,30], reduces DNA end mobility and ability to pair before the formation of a translocation [42]. Therefore, factors implicated in resection also affect DNA end mobility, which is a prerequisite for the ligation of distant ends.

### The "spatio-temporal" gap model for DSB repair

**Fig 5:**
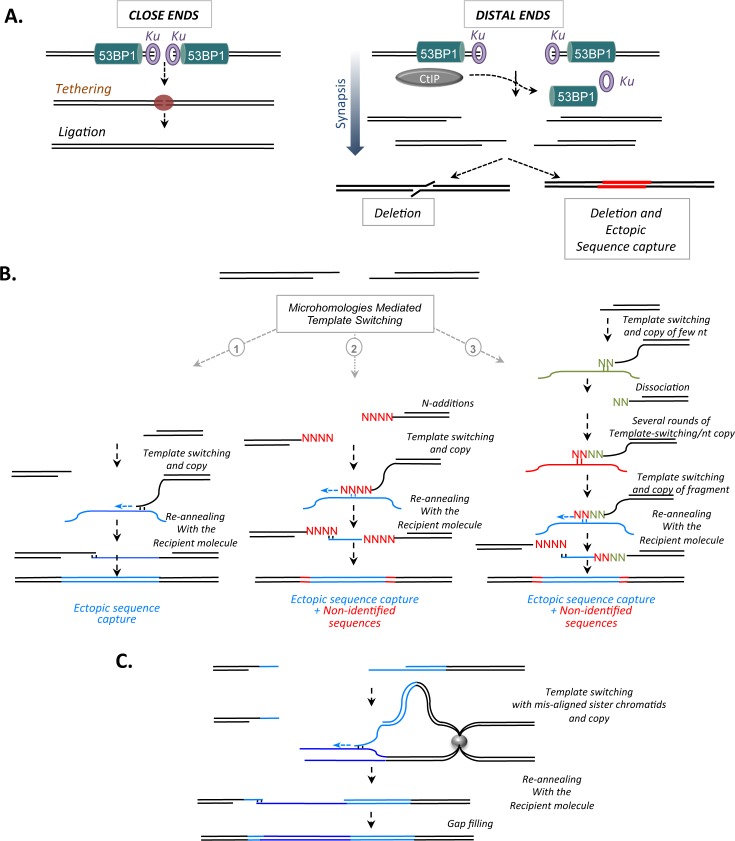
The “spatiotemporal gap” favors ectopic chromosomal sequences. **A.** Close DSEs are immediately tethered and then ligated. Distant DSBs require synapsis to give space and time for dissociation of KU and 53BP1 from DNA ends and for CtIP to intrude and act during this “spatiotemporal gap”. This favors non-conservative events, among them ectopic sequence capture (red). **B.** Hypothetical model for chromosome sequence capture through micro-homology-mediated template switching (MMts). Upper panel: synapsis of distant DSEs. Lower panels: 1- micro-homology template switching (MMts) without unidentified sequences leading to ectopic chromosomal sequence (ECS) capture (in blue); 2- N-additions (in red) by non-template polymerases 3- successive round of MMTS resulting in unidentified sequences (in red and green) at the borders of inserted sequence. **C.** Sequence homology-independent microhomologies-mediated unequal sister chromatid exchange through MMts. Blue lines: reporter sequences. Grey sphere: centromere.

The present data can be unified in the model shown in Fig 5A. Partial duplication of the EJ reporter and ECS capture requires the association of both DSE resection and distance. Indeed, distance creates a “spatiotemporal gap," giving time and space for CtIP to initiate DNA end resection. Both 53BP1 and KU have been proposed to protect DNA ends from degradation. Indeed, with the substrate used here, the absence of KU also increased non-conservative end-joining and long deletions [2,3]. In addition, ablation of the KU70-KU80 heterodimer, which is involved in the tethering of the two DSEs of one DSB, consistently leads to increased mobility of the DSE and genome rearrangement [43]. Therefore, both 53BP1 and KU should protect DSEs. However, for close ends, the absence of 53BP1 should be compensated by the proximity of the two ends, which should permit rapid joining. In addition, close ends favor the tethering of the two ends by KU. This situation does not provide enough time and space for nucleases to attack the DNA extremities, even in the absence of 53BP1. With distant DSEs, the synapsis of the two ends is first required, even with such a short distance as 3.2 kb. In this situation, the tethering of the two ends prior to ligation cannot pre-exist, even in the presence of KU. Therefore, the synapsis step provides space and time for CtIP to initiate resection, resulting in complex sealing patterns. The absence of 53BP1, which counteracts CtIP, increases such events.

Upon replication stress, micro-homology-mediated rearrangements have been proposed in a process called MMBIR/FoSTeS [16]. In yeast, template switching between repeat sequences also generates rearrangements [18]. Associating the present data with the above models, an additional attractive hypothetical model speculates that single-stranded DNA tails generated by CtIP favor microhomology-mediated template switching (MMts), initiating copying of ECSs (Fig 5B). 53BP1 protects against such events. Unidentified N-additions, by increasing ssDNA tail length, can enhance the probability of finding micro-homology to anneal. Different polymerases are able to promote non-template N-additions; for example, in the course of V(D)J recombination [44]. Alternatively, unidentified sequences might result from several successive rounds of annealing/copying of few nucleotides on different templates. In addition, the two aforementioned processes might also cooperate to generate unidentified sequences at the DNA capture borders. Then, similarly to SDSA (synthesis dependent strand annealing) (see [1])), flip back to the recipient molecule by micro-homology annealing with the acceptor molecule, results in ECS capture (Fig 5B). In one particular case of this model, MMts with a misaligned sister chromatid should result in partial duplication of the reporter sequence through sequence homology-independent microhomologies-mediated unequal sister-chromatid exchanges (Fig 5C).

Physiological joining of distant DSEs occurs in V(D)J and class switch recombination and therefore should be highly controlled. For instance, in V(D)J recombination, the cleavage happens after the synapsis has brought the involved sequences close together, thus protecting against potential genome instability generated by the synapsis of distant broken ends. In contrast, replication stress generates unscheduled single-ended DSBs. Consequently, EJ of replication-stress-induced DSEs necessarily involves distant DSEs. Importantly, replication stress has been proposed to act during early stages of malignancy [45,46]. Interestingly, using the substrates described here, we have reported that the cohesin complex protects against the joining of distant ends (but does not inhibit the joining of close ends), specifically in the S phase, thus preventing genetic rearrangements generated by the joining of replication stress-induced double-strand ends [14]. Consistent with the present data, we have previously reported that DNA end mobility generated by cohesin complex ablation also increases the occurrence of long insertions at sites of distant DSE rejoining (3.1% in control cells *vs*. 9.7% in RAD21-depleted cells) [14]. Thus, these data suggest that the substrate used here with distant I-SceI-induced DSBs mimics, at least in part, some features of the joining of distant single-ended DSBs generated by replication stress. The present data show that unscheduled EJ of distant DSEs can yield potentially deleterious genome rearrangements at the repair junction, involving a complex mix/cooperation of different DNA processes. CtIP is required at the initiation of homologous recombination and sister-chromatid exchanges [36], which allows for the restart of arrested replication forks, thereby promoting genome stability maintenance. However, we show here that CtIP is a two-edge sword, jeopardizing genome stability at the joining of distant DSEs. Therefore, by counteracting CtIP, 53BP1 plays a pivotal role in genome stability maintenance of unscheduled DSBs.

## Materials and Methods

### DNA manipulations

All DNA manipulations were performed as previously described [47].

### Cells

The cell lines were derived from SV40-transformed GM639 human fibroblasts and were cultured in DMEM supplemented with 10% fetal calf serum (FCS) and 2 mM glutamine and were incubated at 37°C in 5% CO_2_. Linearized NHEJ reporters were electroporated into the cells, and individual clones were selected using blasticidin (5 μg/mL) or neomycin (500 μg/ml).

### Transfection

The meganuclease I-SceI was expressed by transient transfection of the expression plasmid pCMV-HA-I-SceI (47) with Jet-PEI, following the manufacturer’s instructions (Polyplus transfection, Illkirch, France). The expression of HA-tagged I-SceI was verified by Western blotting. For silencing experiments, 50,000 cells were seeded 1 day before transfection, which was carried out using 20 nmol of onTarget plus SMARTpool for human TP53BP1 (Dharmacon, Chicago, IL, USA), CtIP siRNA (5'- GCUAAAACAGGAACGAAUC -3') and/or control siRNA (SR-CL000-005, Eurogentec, Angers, France; 5’ AUGAACGUGAAUUGCUCAA -3’, #019317273, Eurofins, Ebersberg, Germany) and INTERFERin following the manufacturer’s instructions (Polyplus Transfection, Illkirch, France). Forty-eight hours later, the cells were transfected with the pCMV-HA-I-SceI expression plasmid.

### Measure of End Joining efficiency by FACS

After transfection with the pCMV-HA-I-SceI plasmid and incubation for 72 hours, the cells were collected in PBS and 50 mM EDTA, pelleted and fixed with 2% paraformaldehyde for 20 minutes. The percentage of GFP-expressing cells was scored by FACS analysis using a BD Accuri C6 flow cytometer (BD, Franklin Lakes, NJ, USA). The percentage of CD4-expressing cells was measured after incubation for 10 minutes with 1 μl of anti-CD4 antibody coupled to Alexa 647 (rat isotype, RM4-5, Pharmingen, San Diego, CA, USA). For each cell line, at least 3 independent experiments were performed, and HA-I-SceI expression and efficiency of silencing was verified each time by Western blot.

### Western blotting

For western blot analysis, the cells were lysed in buffer containing 20 mM Tris HCl (pH 7.5), 1 mM Na_2_EDTA, 1 mM EGTA, 150 mM NaCl, 1% (w/v) NP40, 1% sodium deoxycholate, 2.5 sodium pyrophosphate, 1 mM β-glycerophosphate, 1 mM NA_3_VO_4_ and 1 μg/ml leupeptin supplemented with complete mini protease inhibitor (Roche, Mannheim, Germany). Denatured proteins (20–40 μg) were electrophoresed in 9% SDS-PAGE gels, transferred onto a nitrocellulose membrane and probed with the specific antibodies anti-HA (MMS-101R, Covance, Berkeley, CA), anti-53BP1 (#4937, Cell Signaling, Danvers, MA, USA), anti-CtIP (rabbit, courtesy of Dr. R. Baer), and anti αTubulin (#T5168, Sigma Aldrich, Munich, Germany). Immunoreactivity was visualized using an enhanced chemiluminescence detection kit (ECL, Pierce).

### Junction sequence analysis

We amplified the junction sequences by PCR of genomic DNA using the primers CMV-6 (5'-TGGTGATGCGGTTTTGGC-3’) and CD4-int (5'-GCTGCCCCAGAATCTTCCTCT-3'). The PCR products were cloned with a TOPO PCR cloning kit (Invitrogen Life Technologies) and sequenced (GATC Biotech, Konstanz, Germany and Eurofins, Ebersberg, Germany). For each sample, 2 to 5 experiments were pooled in the sequencing data. In each of these experiments, HA-I-SceI expression, and efficiency of silencing were verified by Western blot.

### Identification of insertions

Insertions were blasted using the BLAST program of the National Centre of Biotechnology Information (National Institutes of Health, Bethesda MD, USA). Insertions were blasted to the end joining reporter, the I-SceI expression plasmid, the mitochondrion genome, the human genome (*Homo sapiens*, taxid: 9606), human ALU repeat elements and the nucleotide collection using megablast and discontinuous megablast. Sequences identified as “non-templated nucleotides” were identified by neither of these searches.

### Statistical Analysis

Statistical analyses (Mann-Whitney tests for the size of insertions and t-test for the frequency of insertions and the ratio of conservative *vs*. non-conservative repair) were performed using GraphPad Prism 3.0 (GraphPad Software).

## Supporting Information

S1 Supplementary information Substrates (DOCX)

S2 Supplementary information Sequences of end-joining junctions of close versus distant DSEs. (DOCX)

S3 Supplementary information Impact of CtIP *versus* 53BP1 on close and distant DSEs (DOCX)

S4 Supplementary information Insertions at distant DSEs (CD4-3200bp) (DOCX)

S5 Supplementary information Insertions at close ends (34 bp) (DOCX)

## References

[1] Haber JE. Genome stability DNA repair and recombination. Garland Science, Talor and Francis Group, New York and London; 2014.

[2] Guirouilh-Barbat J, Huck S, Bertrand P, Pirzio L, Desmaze C, Sabatier L, et al Impact of the KU80 pathway on NHEJ-induced genome rearrangements in mammalian cells. Mol Cell. 2004;14: 611–623. 10.1016/j.molcel.2004.05.008 15175156

[3] Guirouilh-Barbat J, Rass E, Plo I, Bertrand P, Lopez BS. Defects in XRCC4 and KU80 differentially affect the joining of distal nonhomologous ends. Proc Natl Acad Sci U S A. 2007;104: 20902–7. 10.1073/pnas.0708541104 18093953 PMC2409239

[4] Betermier M, Bertrand P, Lopez BS. Is non-homologous end-joining really an inherently error-prone process? PLoS Genet. 2014 p. e1004086 10.1371/journal.pgen.1004086 24453986 PMC3894167

[5] Grabarz A, Barascu A, Guirouilh-Barbat J, Lopez BS. Initiation of DNA double strand break repair: signaling and single-stranded resection dictate the choice between homologous recombination, non-homologous end-joining and alternative end-joining. Am J Cancer Res. United States; 2012 pp. 249–268. PMC3365807 22679557

[6] McVey M, Lee SE. MMEJ repair of double-strand breaks (director’s cut): deleted sequences and alternative endings. Trends Genet. 2008 pp. 529–538. 10.1016/j.tig.2008.08.007 18809224 PMC5303623

[7] Bennardo N, Cheng A, Huang N, Stark JM. Alternative-NHEJ is a mechanistically distinct pathway of mammalian chromosome break repair. PLoS Genet. 2008 p. e1000110 10.1371/journal.pgen.1000110 18584027 PMC2430616

[8] Xie A, Kwok A, Scully R. Role of mammalian Mre11 in classical and alternative nonhomologous end joining. Nat Struct Mol Biol. 2009;16: 814–8. 10.1038/nsmb.1640 19633669 PMC2730592

[9] Deriano L, Stracker TH, Baker A, Petrini JHJ, Roth DB. Roles for NBS1 in alternative nonhomologous end-joining of V(D)J recombination intermediates. Mol Cell. Elsevier Ltd; 2009;34: 13–25. 10.1016/j.molcel.2009.03.009 19362533 PMC2704125

[10] Rass E, Grabarz A, Plo I, Gautier J, Bertrand P, Lopez BS. Role of Mre11 in chromosomal nonhomologous end joining in mammalian cells. Nat Struct Mol Biol. 2009 pp. 819–824. 10.1038/nsmb.1641 19633668

[11] Saintigny Y, Delacote F, Vares G, Petitot F, Lambert S, Averbeck D, et al Characterization of homologous recombination induced by replication inhibition in mammalian cells. EMBO J. 2001 pp. 3861–3870. 10.1093/emboj/20.14.3861 11447127 PMC125539

[12] Magdalou I, Lopez BS, Pasero P, Lambert SA. The causes of replication stress and their consequences on genome stability and cell fate. [Internet]. Semin Cell Dev Biol. 2014 pp. 154–164. 10.1016/j.semcdb.2014.04.035 24818779

[13] Gelot C, Magdalou I, Lopez BS. Replication stress in Mammalian cells and its consequences for mitosis. Genes (Basel). 2015;6: 267–98. 26010955 10.3390/genes6020267 PMC4488665

[14] Gelot C, Guirouilh-Barbat J, Le Guen T, Dardillac E, Chailleux C, Canitrot Y, et al The Cohesin Complex Prevents the End Joining of Distant DNA Double-Strand Ends. Mol Cell. 2016;61: 15–26. 10.1016/j.molcel.2015.11.002 26687679

[15] Hastings PJ, Ira G, Lupski JR. A microhomology-mediated break-induced replication model for the origin of human copy number variation. PLoS Genet. 2009 p. e1000327 10.1371/journal.pgen.1000327 19180184 PMC2621351

[16] Zhang F, Khajavi M, Connolly AM, Towne CF, Batish SD, Lupski JR. The DNA replication FoSTeS/MMBIR mechanism can generate genomic, genic and exonic complex rearrangements in humans. Nat Genet. 2009 pp. 849–853. 10.1038/ng.399 19543269 PMC4461229

[17] Zhang F, Carvalho CM, Lupski JR. Complex human chromosomal and genomic rearrangements. Trends Genet. England; 2009 pp. 298–307. 10.1016/j.tig.2009.05.005 PMC4464790 19560228

[18] Anand RP, Tsaponina O, Greenwell PW, Lee C-S, Du W, Petes TD, et al Chromosome rearrangements via template switching between diverged repeated sequences. Genes Dev. 2014;28: 2394–406. 10.1101/gad.250258.114 25367035 PMC4215184

[19] Cary RB, Peterson SR, Wang J, Bear DG, Bradbury EM, Chen DJ. DNA looping by Ku and the DNA-dependent protein kinase. Proc Natl Acad Sci U S A. 1997 pp. 4267–4272. 9113978 10.1073/pnas.94.9.4267 PMC20711

[20] Weterings E, Van Gent DC. The mechanism of non-homologous end-joining: A synopsis of synapsis. DNA Repair. 2004 pp. 1425–1435. 10.1016/j.dnarep.2004.06.003 15380098

[21] Shao Z, Davis AJ, Fattah KR, So S, Sun J, Lee K-JJ, et al Persistently bound Ku at DNA ends attenuates DNA end resection and homologous recombination. DNA Repair (Amst). 2012;11: 310–6. 22265216 10.1016/j.dnarep.2011.12.007 PMC3297478

[22] DeFazio LG, Stansel RM, Griffith JD, Chu G. Synapsis of DNA ends by DNA-dependent protein kinase. Embo J. 2002 pp. 3192–3200. 10.1093/emboj/cdf299 12065431 PMC126055

[23] Soutoglou E, Dorn JF, Sengupta K, Jasin M, Nussenzweig A, Ried T, et al Positional stability of single double-strand breaks in mammalian cells. Nat Cell Biol. 2007 pp. 675–682. 10.1038/ncb1591 17486118 PMC2442898

[24] Yan CT, Boboila C, Souza EK, Franco S, Hickernell TR, Murphy M, et al IgH class switching and translocations use a robust non-classical end-joining pathway. Nature. 2007;449: 478–82. 10.1038/nature06020 17713479

[25] Boboila C, Jankovic M, Yan CT, Wang JH, Wesemann DR, Zhang T, et al Alternative end-joining catalyzes robust IgH locus deletions and translocations in the combined absence of ligase 4 and Ku70. Proc Natl Acad Sci. 2013;107: 3034–3039. 10.1073/pnas.0915067107 PMC2840344 20133803

[26] McVey M, Radut D, Sekelsky JJ. End-joining repair of double-strand breaks in Drosophila melanogaster is largely DNA ligase IV independent. Genetics. 2004;168: 2067–2076. 10.1534/genetics.104.033902 15611176 PMC1448732

[27] Weinstock DM, Brunet E, Jasin M. Formation of NHEJ-derived reciprocal chromosomal translocations does not require Ku70. Nat Cell Biol. 2007. 10.1038/ncb1624 PMC3065497 17643113

[28] Simsek D, Jasin M. Alternative end-joining is suppressed by the canonical NHEJ component Xrcc4-ligase IV during chromosomal translocation formation. Nat Struct Mol Biol. Nature Publishing Group; 2010;17: 410–6. 10.1038/nsmb.1773 20208544 PMC3893185

[29] Ghezraoui H, Piganeau M, Renouf B, Renaud J-B, Sallmyr A, Ruis B, et al Chromosomal translocations in human cells are generated by canonical nonhomologous end-joining. Mol Cell. 2014;55: 829–42. 10.1016/j.molcel.2014.08.002 25201414 PMC4398060

[30] Grabarz A, Guirouilh-Barbat JJ, Barascu AA, Pennarun GG, Genet D, Rass E, et al A role for BLM in double-strand break repair pathway choice: prevention of CtIP/Mre11-mediated alternative nonhomologous end-joining. Cell Rep. 2013;5: 21–8. 10.1016/j.celrep.2013.08.034 24095737

[31] Beck C, Boehler C, Guirouilh Barbat J, Bonnet ME, Illuzzi G, Ronde P, et al PARP3 affects the relative contribution of homologous recombination and nonhomologous end-joining pathways. Nucleic Acids Res. 2014 pp. 5616–5632. 10.1093/nar/gku174 24598253 PMC4027158

[32] Barton O, Naumann SC, Diemer-Biehs R, Künzel J, Steinlage M, Conrad S, et al Polo-like kinase 3 regulates CtIP during DNA double-strand break repair in G1. J Cell Biol. 2014;206: 877–94. 10.1083/jcb.201401146 25267294 PMC4178966

[33] Bothmer A, Robbiani DF, Di Virgilio M, Bunting SF, Klein IA, Feldhahn N, et al Regulation of DNA end joining, resection, and immunoglobulin class switch recombination by 53BP1. Mol Cell. 2011 pp. 319–329. 10.1016/j.molcel.2011.03.019 PMC3142663 21549309

[34] Guirouilh-Barbat J, Huck S, Bertrand P, Pirzio L, Desmaze C, Sabatier L, et al Impact of the KU80 pathway on NHEJ-induced genome rearrangements in mammalian cells. Mol Cell. 2004 pp. 611–623. 10.1016/j.molcel.2004.05.008 15175156

[35] Guirouilh-Barbat J, Rass E, Plo I, Bertrand P, Lopez BS. Defects in XRCC4 and KU80 differentially affect the joining of distal nonhomologous ends. Proc Natl Acad Sci U S A. 2007 pp. 20902–20907. 10.1073/pnas.0708541104 18093953 PMC2409239

[36] Grabarz A, Guirouilh-Barbat J, Barascu A, Pennarun G, Genet D, Rass E, et al A role for BLM in double-strand break repair pathway choice: prevention of CtIP/Mre11-mediated alternative nonhomologous end-joining. Cell Rep. 2013 pp. 21–28. 10.1016/j.celrep.2013.08.034 24095737

[37] Bunting SF, Call??n E, Wong N, Chen HT, Polato F, Gunn A, et al 53BP1 inhibits homologous recombination in brca1-deficient cells by blocking resection of DNA breaks. Cell. 2010;141: 243–254. 10.1016/j.cell.2010.03.012 20362325 PMC2857570

[38] Zucman-Rossi J, Legoix P, Victor JM, Lopez B, Thomas G. Chromosome translocation based on illegitimate recombination in human tumors. Proc Natl Acad Sci U S A. 1998 pp. 11786–11791. 9751743 10.1073/pnas.95.20.11786 PMC21718

[39] Piganeau M, Ghezraoui H, De Cian A, Guittat L, Tomishima M, Perrouault L, et al Cancer translocations in human cells induced by zinc finger and TALE nucleases. Genome Res. 2013 pp. 1182–1193. 10.1101/gr.147314.112 23568838 PMC3698511

[40] Dion V, Kalck V, Horigome C, Towbin BD, Gasser SM. Increased mobility of double-strand breaks requires Mec1, Rad9 and the homologous recombination machinery. Nat Cell Biol. 2012;14: 502–509. 10.1038/ncb2465 22484486

[41] Miné-Hattab J, Rothstein R. Increased chromosome mobility facilitates homology search during recombination. Nat Cell Biol. 2012;14: 510–517. 10.1038/ncb2472 22484485

[42] Roukos V, Voss TC, Schmidt CK, Lee S, Wangsa D, Misteli T. Spatial dynamics of chromosome translocations in living cells. Science. 2013;341: 660–4. 10.1126/science.1237150 23929981 PMC6324928

[43] Soutoglou E, Dorn JF, Sengupta K, Jasin M, Nussenzweig A, Ried T, et al Positional stability of single double-strand breaks in mammalian cells. Nat Cell Biol. Nature Publishing Group; 2007;9: 675–82. 10.1038/ncb1591 17486118 PMC2442898

[44] Jung D, Alt FW. Unraveling V(D)J recombination; insights into gene regulation. Cell. 2004 pp. 299–311. 10.1016/s0092-8674(04)00039-x 14744439

[45] Gorgoulis VG, Vassiliou L V, Karakaidos P, Zacharatos P, Kotsinas A, Liloglou T, et al Activation of the DNA damage checkpoint and genomic instability in human precancerous lesions. Nature. 2005 pp. 907–913. 10.1038/nature03485 15829965

[46] Bartkova J, Horejsi Z, Koed K, Kramer A, Tort F, Zieger K, et al DNA damage response as a candidate anti-cancer barrier in early human tumorigenesis. Nature. 2005 pp. 864–870. 10.1038/nature03482 15829956

[47] Ausubel FM, Brent R, Kingston RE, Moore DD, Seidman JG, Smith JA, et al Current Protocols in Molecular Biology John Wiley & Sons, Inc.,Boston; 1999.

[48] Xie A, Kwok A, Scully R. Role of mammalian Mre11 in classical and alternative nonhomologous end joining. Nat Struct Mol Biol. 2009 pp. 814–818. 10.1038/nsmb.1640 19633669 PMC2730592

