# Supplementary material for: 53BP1 Protects against CtIP-Dependent Capture of Ectopic Chromosomal Sequences at the Junction of Distant Double-Strand Breaks
Source: PLoS Genet. 2016 Oct 31;12(10):e1006230. doi: 10.1371/journal.pgen.1006230 (PMC5087911; doi:10.1371/journal.pgen.1006230)
Supplement: S1 Supplementary information — (DOCX) [file pgen.1006230.s001.docx]

***SUPPLEMENTARY INFORMATIONS***

***S1***

**Substrates**

***S1-I. Substrate with distal ends (example of the CD4-3200 substrate)***

**A/**

**
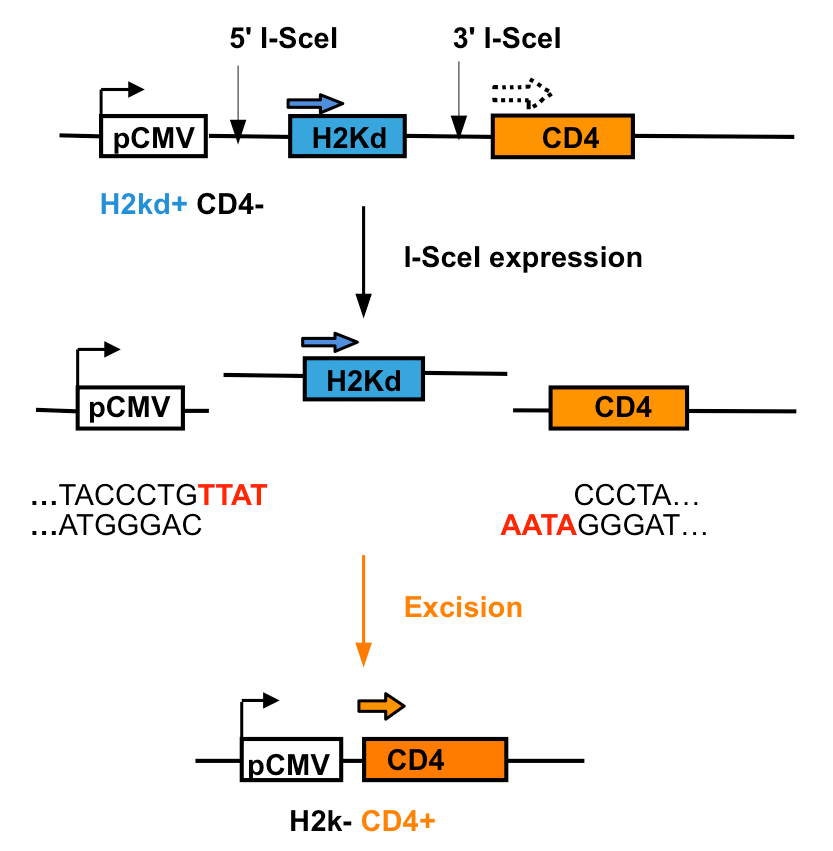
**

**B/**


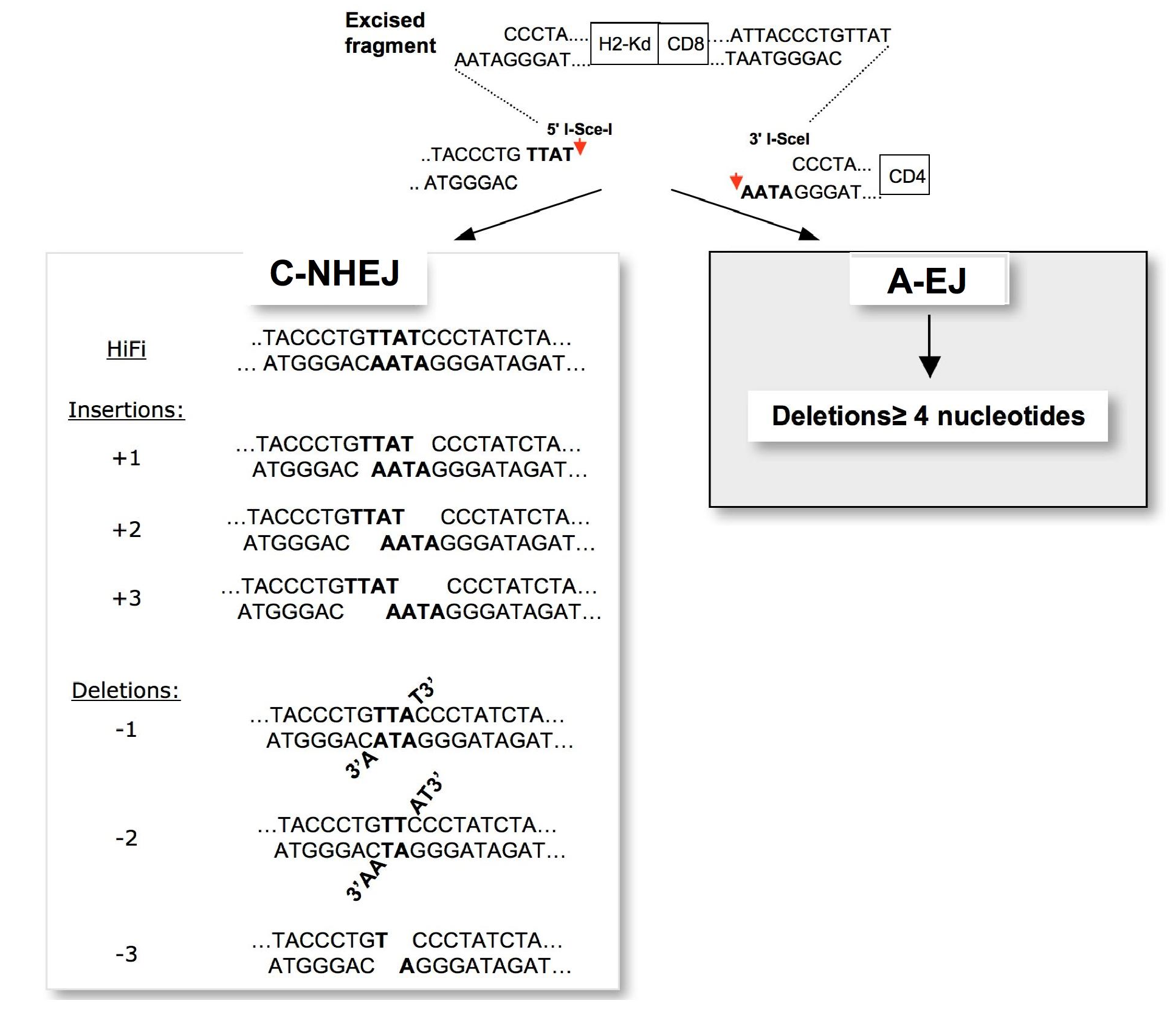


**Figure S1-I A/** The intrachromosomal end-joining substrate with distal ends (example of the CD4-derived substrate). In the absence of expression of the meganuclease I-SceI, CD4 is not expressed as it is too far from the promoter. Two I-SceI cleavage sites flank the fragment containing H2Kd. After cleavage by I-SceI, rejoining of the DNA ends leads to the excision of the internal H2Kd fragment and the expression of CD4. These events were measured by FACS and the resealed junctions were amplified by PCR and sequenced. **B.** Outcome with the CD4-3200bp substrate. Examples of end-joining intermediates in C-NHEJ (left panel), which are KU/Lig4-dependant and A-EJ (right panel), which are KU/Lig4-independant. Upper panel: the structure of the I-SceI cleavage site (bold type indicates the four 3’-protruding nucleotides generated by I-SceI cleavage). C-NHEJ and A-EJ differ in the junction patterns that occur after the resealing of the DNA ends. Whereas C-NHEJ uses the four 3’-protruding nucleotides (3’-Pnt) generated by I-SceI cleavage, A-EJ is characterized by deletions at the junctions of 3’ protrusions of at least four, but usually more, nucleotides.C-NHEJ uses the four 3’-Pnt; it is able to join both fully and non-fully cohesive ends and can generate imperfect annealing. These intermediates are then processed for gap filling and mismatch repair, leading either to error-free end-joining (HiFi, perfect annealing) or the deletion or insertion of 1 to 3 nucleotides at the cleavage sites according to the intermediates. Such events have not been observed in *KU*- or *XRCC4*-deficient cells. In *ku-* or *xrcc4*-deficient cells (which therefore exclusively use A-EJ), the use of the 3’-Pnt disappears, HiFi events are extremely rare, and 1- to 3-nt insertions or deletions are never observed; in contrast, the deletion of at least the four 3’-Pnt (and generally more extended regions) is observed (Grabarz et al., 2013; Guirouilh-Barbat et al., 2004, 2007; Rass et al., 2009).

In conclusion, the following generalities apply:

- The use of at least one of the 3’-Pnt corresponds to C-NHEJ.
- Deletions of at least the 4 3’-Pnt at the junctions (generated by I-SceI) are a hallmark of A-EJ

***S1-II. Substrate with close ends (example of the GFP-34bp substrate)***


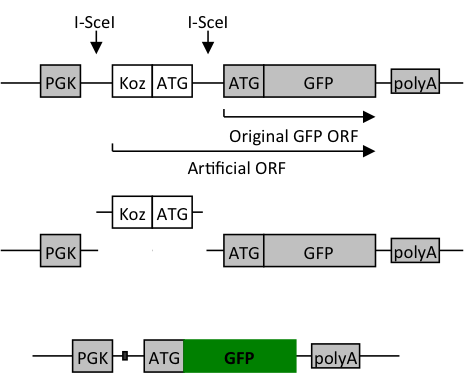


**Figure S1-II.** The intrachromosomal end-joining substrate with close ends (example of the GFP-derived substrate). The translation of a wild-type copy of the gene encoding enhanced green fluorescent protein ('*GFP*') is suppressed by an upstream, out-of-frame translation start site ('Koz-ATG') flanked by two I-SceI sites. Cleavage by I-SceI releases Koz-ATG, and ligation of the DNA ends allows translation of *GFP* in the correct frame. PGK: phosphoglycerate kinase. “Koz-ATG”: an artificial Kozak-ATG translation start site. ORF: open reading frame. PolyA: polyadenylation signal (Xie et al., 2009).

**References**

Grabarz, A., Guirouilh-Barbat, J., Barascu, A., Pennarun, G., Genet, D., Rass, E., Germann, S.M., Bertrand, P., Hickson, I.D., and Lopez, B.S. (2013). A role for BLM in double-strand break repair pathway choice: prevention of CtIP/Mre11-mediated alternative nonhomologous end-joining. Cell Rep *5*, 21–28.

Guirouilh-Barbat, J., Huck, S., Bertrand, P., Pirzio, L., Desmaze, C., Sabatier, L., and Lopez, B.S. (2004). Impact of the KU80 pathway on NHEJ-induced genome rearrangements in mammalian cells. Mol Cell *14*, 611–623.

Guirouilh-Barbat, J., Rass, E., Plo, I., Bertrand, P., and Lopez, B.S. (2007). Defects in XRCC4 and KU80 differentially affect the joining of distal nonhomologous ends. Proc Natl Acad Sci U S A *104*, 20902–20907.

Rass, E., Grabarz, A., Plo, I., Gautier, J., Bertrand, P., and Lopez, B.S. (2009). Role of Mre11 in chromosomal nonhomologous end joining in mammalian cells. Nat Struct Mol Biol *16*, 819–824.

Xie, A., Kwok, A., and Scully, R. (2009). Role of mammalian Mre11 in classical and alternative nonhomologous end joining. Nat Struct Mol Biol *16*, 814–818.
