# Supplementary material for: 53BP1 Protects against CtIP-Dependent Capture of Ectopic Chromosomal Sequences at the Junction of Distant Double-Strand Breaks
Source: PLoS Genet. 2016 Oct 31;12(10):e1006230. doi: 10.1371/journal.pgen.1006230 (PMC5087911; doi:10.1371/journal.pgen.1006230)
Supplement: S2 Supplementary information — (DOCX) [file pgen.1006230.s002.docx]

***S2***

**Sequences of end-joining junctions of close *versus* distal DSEs.**

**Control siRNA, Distant DNA ends (CD4-3200bp) GC92 cells**

Total number of sequences: 146

- **HiFi: 69/190 (36%)**

CTAGAGCAACACGGAAGGA**ATTACCCTGTTATCCCTA**TCTAGATATGAAA 47X

CTAGAGCAACACGGAAGGA**ATTACCCTGT------CCCTA**TCTAGATATGAAA 5X

CTAGAGCAACACGGAAGGA**ATTACCCTGT----TCCCTA**TCTAGATATGAAA 7X

CTAGAGCAACACGGAAGGA**ATTACCCTGT--ATCCCTA**TCTAGATATGAAA 8X

CTAGAGCAACACGGAAGGA**ATTACCCTG----ATCCCTA**TCTAGATATGAAA 1X

CTAGAGCAACACGGAAGGA**ATTACCCTGTT--TCCCTA**TCTAGATATGAAA 1X

- **Insertion: 7/190 (4%)**

CTAGAGCAACACGGAAGGA**ATTACCCTGTTATATCCCTA**TCTAGATATGAAA +2

CTAGAGCAACACGGAAGGA**ATTACCCTGTTATTATCCCTA**TCTAGATATGAAA +3

GCAACACGGAAGGA**ATCGCGCATAATCGATATTACCCTGTTATCCCTA**TCTA +16

CTAGAGCAACACGGAAGGA**ATTACCCTGTTTATCCCTATCTA**GATATGAAA +1

CTAGAGCAACACGGAAGGA**ATTACCCTGTTATC**---ins148----- **CCTATCTA**GATA +148

CTAGAGCAACACGGAAGGA**ATTACCCTGTTATTATCCCTA**TCTAGATATGAAA +3

CTAGAGCAACACGGAAGGA**ATTACCCTGTTATCC**----ins 151--- **CTA**TCTAGATAT +151

- **Deletion: 87/190 (46%)**

CTAGAGCAACACGGAAGGA**ATTA**------del 8------**TCCCTA**TCTAGATATGAAA -8

CTAGAGCAACACGGAAGGA**ATTACCCT**-------del 9-------**A**TCTAGATATGAAA -9

**CTAGAGCAACACGGAAGGAATTACC**---------del 10----**TA**TCTAGATATGAAA -10

AAAATGTCGTAACAACTCCGCCCCAT------del 166-------CTAGATATGAAATC -166

CTAGCGCTC------------------------del 38--------------------------------TAGATATGAAA -38

CTAGAGCAACACGGAAGGA-----------del 24-------------------------------TATGAAA -24

CTAGAGCAACACGGAAGGA**ATTA**-------del 8-----**TCCCTA**TCTAGATATGAAA -8

CTAGAGCAACACGGAAGGA**ATTA**------------del 14-----------TCTAGATATGAAA -14

CTAGAGCAACACGGAAGGAATTACCCT------------del 22---------------------------A -22

CTAGAGCAACACGGAAGGA**ATTA**-------del 14----------------TCTAGATATGAAA -14

CTAGAGCAACACGGAAGGAATTACCCT----del 84-------CACGGAAGGAATTA -84

CTAGAGCAACACGGAAGGA**ATTACCCT**----del 9---------**A**TCTAGATATGAAA -9

CTAGAGCAACACGGAAGGA**ATTACCCT**------del 9--------**A**TCTAGATATGAAA -9

CTAGAGCAACACGGAAGGA**ATTACCCT**------del 9--------**A**TCTAGATATGAAA -9

CTAGAGCAACACGGAAGGA**ATTACCCT**------del 9 -------**A**TCTAGATATGAAA -9

CTAGAGCAACACGGAAGGA**ATTACCCT**------del 9--------**A**TCTAGATATGAAA -9

CTAGAGCA ACACGGAAGG**AATTACC**—del 3—**TTATCCCTA**TCTAGATATGAAA -3

CTAGAGCAACACGGAAGGA**ATTACCCTG**-----del 4---**CCCTA**TCTAGATATGAAA -4

CTAGAGCAACACGGAAGGA**ATTACCCTGTTATCC**-del1-**TA**TCTAGATATGAAA -1

CTAGAGCAACACGGAAGGA**ATTACCCTGTTATCC**-del1-**TA**TCTAGATATGAAA -1

CTAGAGCAACACGGAAGGA**ATTACCCTGTTAT**---del 4-----**A**TCTAGATATGAAA -4

CTAGAGCAACACGGAAGGA**ATTACCCTGTTA**-------del 288-----------AGGGAAGA -288

CTAGAGCAACACGGAAGGA**ATTACCCT**-----del 9--------------**A**TCTAGATATGAAA -9

CTAGAGCAACACGGAAGGA**ATTACCCT**-----del 9--------------**A**TCTAGATATGAAA -9

CTAGAGCAA CACGGAAGGA**AT**------del 11---------------**CCCTA**TCTAGATATGAAA -11

CTAGAGCAACACGGAAG-----------------------del 30--------------------------------------AAA -30

CTAGAGCAACACGGAAGGAATTACCCTGTTATC---del 6------------TAGATATGAAA -6

CTAGAGCAACACGGAAGGA**ATT**----del 9-----------------**TCCCTA**TCTAGATATGAAA -9

CTAGAGCAACACGGAAGGA**ATTACCC**-----del 7------------**CCTA**TCTAGATATGAAA -7

CTAGAGCAACACGGAAGGA**ATTACCCTGTTATC**-----del 6----------TAGATATGAAA -6

CTAGAGCAACACGGAAGGA**ATTACCCT**------del 9----------------**A**TCTAGATATGAAA -9

GTTTGACTCACGGGGATTTCC------------del 262----------------**TA**TCTAGATATGAAA -262

CTAGAGCAACACGGAAGGA**ATTACCCTGTTATC**---del 6-----------TAGATATGAAA -6

CTAGAGCAACACGGAAGGA**ATTACCCT**-------del 9-------------**A**TCTAGATATGAAA -9

CGGTTTGACTCACGGGGATTTCC----------------del 262--------**TA**TCTAGATATGAAA -262

CTAGAGCAACACGGAAGGA**ATTACCCT**----del 3-----**ATCCCTA**TCTAGATATGAAA -3

CTAGAGCAACACGGAA----------------------------------del 21----------TCTAGATATGAAA -21

CTAGAGCAACACGGAAGGA**ATTACCCT**--------del 9-------------**A**TCTAGATATGAAA -9

CTAGAGCAACACGGAAGGA**ATTA**--------del 8-----------**TCCCTA**TCTAGATATGAAA -8

CTAGAGCAACACGGAAGGA**ATTACCCT**-----del 9----------------**A**TCTAGATATGAAA -9

CTAGAGCAACACGGAAGGA**ATTACCCT**-----del 9----------------**A**TCTAGATATGAAA -9

cTAGAGCAACAC------------------------del 42-----------------GCCATGTAGTGTATTGA -42

CTAGAGCAACACGGAAGGA**ATTACCCT**-----del 9----------------**A**TCTAGATATGAAA -9

CTAGAGCAACACGGAAGGA**ATTACCCT**----del 9----------------**A**TCTAGATATGAAA -9

CTAGAGCAACACGGAAGGA**ATTACCCTGTTAT**----del 3----**TA**TCTAGATATGAAA -3

CTAGAGCAACACGGAAGGA**ATTACCCT**---------------del 9-----**A**TCTAGATATGAAA -9

CTAGAGCAACACGGAAGGA**ATTACCCT**---------------del 9-----**A**TCTAGATATGAAA -9

CTAGAGCAACACGGAAGGA**ATTACCCT**--------------del 9------**A**TCTAGATATGAAA -9

CTAGAGCAACACGGAAGGA**ATTA**-----del 8--------------**TCCCTA**TCTAGATATGAAA -8

CTAGAGCAACACGGAAGGA**ATTACC**------------del 12-------------TCTAGATATGAAA -12

CTAGAGCAACACGGAAGGA**ATTACCCTGTTAT**----------del 14-------------------GAAA -14

CTAGAGCAACACGGAAGGA**ATTACCCT**------del 9--------------**A**TCTAGATATGAAA -9

CTAGAGCAACACGGAAGGA**AT**--------del 12----------------**CCTA**TCTAGATATGAAA -12

CTAGAGCAACACGGAAGGA**ATTACCCTGTTATC**---del 6-----------TAGATATGAAA -6

CTAGAGCAACACGGAAGGA**ATTACCCTGT**------del 6--------**TA**TCTAGATATGAAA -6

CTAGAGCAACACGGAAGGA**ATT**-------del 10-------------**CCCTA**TCTAGATATGAAA -10

CTAGAGCAACACGGAAGGA**ATTACCCT**----------del 9----------**A**TCTAGATATGAAA -9

GAGACCCAAGCTGGCTAGC--------------del 57----------------GCCATGTAGTGTATTG -57

CTACCGCTCTAGAGCAACAC-------------del 24----------------------TCTAGATATGAAA -24

CTAGAGCAACACGGAAGGA**ATTACCCT**----------del 9----------**A**TCTAGATATGAAA -9

CTAGAACAACACGGAAGGA**A**-------------del 9--------**TATCCCTA**TCTAGATATGAAA -9

CTAGAGCA------------------------------del 300------------------------------------------------------- -300

CTAGAGCAACACGGA-----------------del 14--------------**TATCCCTA**TCTAGATATGAAA -14

CTAGAGCAACACGGAAGGA**ATTACCCT**----------del 9----------**A**TCTAGATATGAAA -9

CTAGAGCAACACGGAAGG-----------------del 330 ------------GGAATCAGCAGAACTGC -330

CTAGAGCAACACGGAAGGA**ATTACCCTGTTAT**--------del 14-----GAAATCACGCCA -14

CTATAGGGAGACCCAAGCTGGCTA--------------del 42 -------------TCTAGATATGAAA -42

CTAGAGCAACACGGAAGGA**ATTACCCT**------del 9--------------**A**TCTAGATATGAAA -9

CTAGAGCAACACGGAAGGA**ATTA**---------del 14--------------------TCTAGATATGAAA -14

AGCGCTCTAGAGCA-----------------------del 250------CTCTCTTAGGCGCTTGCTGCTGC -250

CTAGAGCAAACGG-------------------------del 41--------------CCATGTAGTGTATTGACCG -41

CTAGAGCAACACGGAAGGA**ATTACCCT**------del 9-------------**A**TCTAGATATGAAA -9

CTAGAGCAACACGGAAGGA**ATTACCCTGTTA**-------del 8-----------TAGATATGAAA -8

CTAGAGCAACACGGAAG------------del 30--------AAATCACGCCATGTAGTGTATTGA -30

CGCCCCATTGACGCAAATG--------------del 316------------GGAAGGACTGGCCAGAGGC -316

CTAGAGCAACACGGAAGGA**AT**------del 8-------------**TATCCCTA**TCTAGATATGAAA -8

CTAGAGCAACACGGAAGGA----------del 16-----------------------**TA**TCTAGATATGAAA -16

GTGGGAGGTCTATATA-------------del 23----------AGAACCCACTGCTTACTGGCTTAT -23

CTAGAGCAACACGGAAGGA**AT**-----------del 15--------------------**A**TCTAGATATGAAA -15

CTAGAGCAACACGGAAGGA**AT**-------------del 23----------ATGAAATCACGCCATGTA -23

CTAGAGCAACACGGAAGGA**ATTACCCT**-------del 9---------**A**TCTAGATATGAAA -9

CTAGAGCAACACGGAAGGA**ATTACCCT**-------del 9---------**A**TCTAGATATGAAA -9

CTAGAGCAACACGGAAGGA**ATTACCCT**-------del 9---------**A**TCTAGATATGAAA -9

CTAGAGCAACACGGAAGGA**ATTACCCT**-------del 9---------**A**TCTAGATATGAAA -9

CTAGAGCAACACGGAAGGA**ATTACCCT**-------del 9---------**A**TCTAGATATGAAA -9

CTAGAGCAACACGGAAGGA**ATTACCCTGT**-------del 9---------CTAGATATGAAA -9

CTAGAGCAACACGGAAGGAAT-------del 8--------**TATCCCTA**TCTAGATATGAAA -8

- **Deletion / Insertion: 27/190 (14%)**

AAAATGTCGTAA------del 186/ins 16----ATCTAGATATGAAATCACGCCATGTA -186+16

CTAGAGCAACACGGAAGGAATT----del 11/ins 5-----CCTATCTAGATATGAAA -11+5

CTAGAGCAACACGGAAGGA**ATTACCCTGT**-----del 264/ins 334------CTGTCAC -264+334

CTAGAGCAACACGGAAGGA**ATTACCCTGTTATC**—del 9/ins 3 ----ATATGAAA -9+3

CTAGAGCAACACGGAAG---------del 18/ins 2----------------TATCTAGATATGAAA -18+2

AGCTGGCTAGC----------------del 55/ins 1-------------ACGCCATGTAGTGTATTGA -55+1

CTAGAGCAACACGGAAGGA**ATTACC**----del 4/ins 10------**TA**TCCCTATCTAGA -4+10

CTAGAGCAA CACGGAAGGA**AT**---del 10/ins 11----TCCCTATCTAGATATGAAA -10+11

TGTCGTAACAACTCCGCCCCA---del 172/ins 427------------CCCTATCTAGATAT -172+427

GGTGATGCGGTTTTGGCA----del 301/ins 300---**TATCCCTA**TCTAGATATGAAA -301+300

TGGTGATGCGGTTTTG---del 308/ins 306--------------**CCTA**TCTAGATATGAA -308+306

CTAGAGCAACACGGAAG--------del 16 /ins 2-----------CCTATCTAGATATGAAA -16+2

CTAGCGCTCTAGAGCAACACGGA--------del 20/ins 4----TATCTAGATATGAAA -20+4

TGGTGATGCGGTTTTGGCA------------------del 710/ins 191---------------------------- -710+191

ACTGGCTTATCGAAAT----------------------del 75/ins 2------CTATCTAGATATGAAA -75+2

CTAGAGCAACAC----------------del 169/ins 258----------------GAGTGAAGGAAGG -169+258

TAGAGCAACACG------------del 55/ins13-------GACCGATTCCTTGCGGTCCGAA -55+13

TGGTGATGCGGTTTTGGC--------------------del 710/ins 467------------------------ -710+467

CTAGAGCAA CACGGAAGGAATT--------del 12/ins 7------TATCTAGATATGAAA -12+7

CTAGAGCAACACGGAAGGA---------------del 309/ins 3-------- CGCTGGTGCTG -309+3

TGGTGATGCGGTTTTGGC----------------del 710/ins 88------------------------------------ -710+88

CACGGAAGGA**ATTACCCTGTTA**------del 1/ins 20—CCCTATCTAGATATGAAA -1+20

TGGTGATGCGGTTTTGGC------------------------ del 710/ins 154--------------------------- -710+154

TGGTGATGCGGTTTTGGC------------------------ del 710/ins 154--------------------------- -710+154

CTAGAGCAACACGGAAGGA**ATTA**------del 16/ins 2---------------TAGATATGAAA -16+2

CTAGAGCAACACGGAAGGA---------------------del 20/ins 5--------TAGATATGAAA -20+5

CTAGAGCAACACGGAAGG---------del 14/ins 3---------**CCCTA**TCTAGATATGAAA -14+3

**Control siRNA, Distant DNA ends (CD4-3200bp) GC49 cells**

Total number of sequences: 80

- **HiFi: 18/80 (23%)**

CTAGAGCAACACGGAAGGA**ATTACCCTGTTATCCCTA**TCTAGATATGAAA 16X

CTAGAGCAACACGGAAGGA**ATTACCCTGT-ATCCCTA**TCTAGATATGAAA 2X

- **Insertion: 8/80 (10%)**

CACGGAAGGA**ATTAC**--------ins 33----------**CCTGTTATCCCTA**TCTAGATATG +33

CACGGAAGGA**ATTACCCTGTTA**------ins 18-----**T**CCCTATCTAGATATGAAATC +18

CACGGAAGGA**ATTACCCTGT**------ins 12-------------**TATCCCTA**TCTAGATATGAA +12

CACGGAAGGA**ATTACCCTGT**--------ins 12-----------**TATCCCTA**TCTAGATATGAA +12

CACGGAAGGA**ATTACCCTGTTA**----------ins 18-------**TCCCTA**TCTAGATATGAAA +18

CACGGAAGGAA**TTACCCTGTTA**—ins 4—**TCCCTA**TCTAGATATGAAATCACGC +4

CACGGAAGGAA**TTACCCTGT**-----ins 3----**TATCCCTA**TCTAGATATGAAATCACG +3

CACGGAAGGAA**TTACCCTGTTAT**--------ins 118------------**CCCTA**TCTAGATATGA +118

- **Deletion: 38/80 (47%)**

AGCAACACGGAAGGAA**TTACCCTG**---------------del 262------------AGCTGTCACA -262

AGCAACACGGAAGGAA**TTA**----------del 8---------**TCCCTA**TCTAGATATGAAAT -8

AGCAACACGGAAGGAA**TTA**----------del 8---------**TCCCTA**TCTAGATATGAAAT -8

AGCAACACGGAAGGAA**TTACCCT**----------del 9-----------**A**TCTAGATATGAAAT -9

AGCAACACGGAAGGAA**TTACCCTGT**------del 9--------------CTAGATATGAAAT -9

TAGGGACAACACGGACGGA-------del 9----**TTATCCCTA**TCTAGATATGAAAT -9

CTAGCGCTCTAGAGCAAC-----------del 33------------TATGAAATCACGCCATGTA 33

AGCAACACGGAAGGAA**T**-------del 8-----------**TATCCCTA**TCTAGATATGAAAT -8

AGCAACACGGAAGGAA**TTACC**-------------del 10--------**TA**TCTAGATATGAAAT -10

AGCAACACGGAAGGAA**TTACCCT**------------del 17-----------ATGAAATCACGCC -17

AACTCCGCCCCATTGACGCAAATG-----------del 423----------TCACAACTCCTAGCT -423

TCAATGGGCGTGGATA-----------------del 450---------------------GCCAGAGGCTCAGA -450

ACCAACACGGAAGGAA**TTA**---------------del 14----------------TCTAGATATGAAAT -14

AGCAACACGGAAGGAA**TTACCC**---del 3------**TATCCCTA**TCTAGATATGAAAT -3

TAGGGAGACCCAAGCTGGCT--------------del 155----------------------AGCCCTCATAT -155

AGCAACACGGAAGGAA**TTACCCT**--------------del 9---------**A**TCTAGATATGAAAT -9

AGCAACACGGAAGGAA**TTACCCT**--------------del 9---------**A**TCTAGATATGAAA -9

CTAGCGCTCTAGAGCAACAC----------del 21-----------**CCTA**TCTAGATATGAAAT -21

GCAACACGGAAGGAA**TTACCCT**----del 9--------------------**A**TCTAGATATGAAAT -9

AGCAACACGGAAGGAA----------------del 98----------------TCGAGCTCGCCCGGGGA -98

AGCAACACGGAAGGAA**TTACCCTGT**-----del 9------------------CTAGATATGAAAT -9

GCTGGCTAGCGCTC-------------------del 37-------------------TAGATATGAAATCACGC -37

AGCAACACGGAAGGAA**TTACCCT**------del 9------------------**A**TCTAGATATGAAAT -9

AGCAACACGGAAGGAA**TTACCCT**------del 9------------------**A**TCTAGATATGAAAT -9

AGCAACACGGAAGGAA------------------del 29------------ATCACGCCATGTAGTGTA -29

GGGCGTGGATAGCGGT----------------------------del 293--------AATCACGCCATGTAG -293

AGCAACAAGGAACGAA**TT**------del 9-----------------**TCCCTA**TCTAGATATGAAAT -9

AGCAACACGGAAGGAA**TTACCC**-----del 8----------------**CTA**TCTAGATATGAAAT -8

AGCAACACGGAAGGAA**T**-----del 8----------------**TATCCCTA**TCTAGATATGAAAT -8

AGCAACACGGAAGGAA-----------------del 29----------------ATCACGCCATGTAGTGT -29

GGAAGGAATTACCCTGT----------------del 261--------------AGCTGTCACAACTCCTAG -261

TCAATGGGCGTGGATAGCGGT-------------del 293---------AATCACGCCATGTAGTGT -293

AGCAACACGGAAGGAA**TTAC**---------del 11-----------------**TA**TCTAGATATGAAAT -11

AGCAACACGGAAGGAA**TTACCCT**------del 9------------------**A**TCTAGATATGAAAT -9

AGCAACACGGAAGGAA**TT**-------------del 9------------**TCCCTA**TCTAGATATGAAAT -9

AGCAACACGGAAGGAA**TTAC**-----del 6-------------**ATCCCTA**TCTAGATATGAAAT -6

AGCAACACGGAAGGAA**TTACCCT**-------del 9-----------------**A**TCTAGATATGAAAT -9

AGCAACACGGAAGGAA**TT**---------del 9----------------**TCCCTA**TCTAGATATGAAAT -9

- **Deletion / Insertion: 16/80 (20%)**

AGCAACACGGAA-----------del 19/ins 7----------------------------**TA**TCTAGATATGAAAT -19+7

AGCAAACGGAAGGAA**TTACC**------del 3/ins 14--**TTATCCCTA**TCTAGATATGAAAT -3+14

TTGACTCACGGGGATTTCCAAGTC-------del 338/ins 1------AATTCGAGCTCGCCCGG -338+1

CAACACGGAAGGAATTACCCT-------del 71/ins 3----------ACCCGGTCACCCATTCGA -71+3

CACGGAAGGAA**TTACCCTGTTAT**------del 86/ins 41-------TCGAGCTCGCCCGGGGA -86+41

AATACGACTCACTATAGGGA--------------del 88/ins 24--------GACCGATTCCTTGCGGT -88+24

CACGGAAGGAA**TT**--------------------del 6/ins 6-------**TTATCCCTA**TCTAGATATGAAAT -6+6

AGCAACACGGAAGGAATTA---------del 128/ins 1-------CCTCATATACACACACCTGTG -128+1

AGCAACACGGAAGGAA**TT**-----------del 7/ins 2-------**TATCCCTA**TCTAGATATGAAAT -7+2

AGCAACACGGAAGGAA**TTA**-----del 7/ins 17----------**TATCCCTA**TCTAGATATGAAAT -7+17

CGCTCTAGAGCAACACGGAAGG-------del 298/ins 7-------TCAAGGGAAGACGCTGGTG -298+7

CATCGACGTCAATGGGA---------del226/ins 23---------**TATCCCTA**TCTAGATATGAAAT -226+23

CACGGAAGGA**ATTACC**----------del 5/ins 10---------GTTATCCCTATCTAGATATGAAAT -5+10

GCTCTAGAGCAACACGGA-------del 180/ins 2---------GAAGCAGAGTGAAGGAAGGACT -180+2

CTCTAGAGCAACACGGAAGGA-------del 13/ins 6------------CCCTATCTAGATATGAAAT -13+6

AGCAACACGGAAGGAA**TTACCCT**------del 3/ins 220--**ATCCCTA**TCTAGATATGAAAT -3+220

**Control siRNA, Close DNA ends (CD4-34bp) GCK20 cells**

Total number of sequences: 135

**Conservative Repair : 86/135 (64%)**

- **HiFi: 77/135 (57%)**

CTAGAGCAACACGGAAGGA**ATTACCCTGTTATCCCTA**TCTAGATATGAAA 73X

CTAGAGCAACACGGAAGGA**ATTACCCTGTTA--CCCTA**TCTAGATATGAAA 1X

CTAGAGCAACACGGAAGGA**ATTACCCTGT--ATCCCTA**TCTAGATATGAAA 2X

CTAGAGCAACACGGAAGGA**ATTACCCTGTT----CCCTA**TCTAGATATGAAA 1X

- **Insertion : 9/135 (7%)**

AACACGGAAGGA**ATTACCCTGTTA**----ins 117--- **TCCCTA**TCTAGATATGA +117

AACACGGAAGGA**ATTACCCTGTTA**-----ins 9-------**TCCCTA**TCTAGATATGA +9

AACACGGAAGGA**ATTACCCTGTTATCCCTA**----ins 7---TCTAGATATGAAA +7

AACACGGAAGGA-----ins 34-----**ATTACCCTGTTATCCCTA**TCTAGATATGA +34

AACACGGAAGGA**ATTACCCTGT**-----ins 7------**TATCCCTA**TCTAGATATGA +7

AACACGGAAGGA**ATTACCCTGTTAT**---ins 5-----**CCCTA**TCTAGATATGAAA +5

AACACGGAAGGA**ATTACCCTGT**----ins 7-----**TATCCCTA**TCTAGATATGAAA +7

AACACGGAAGGA------ins 33-----------------**ATTACCCTGTTATCCCTA**TCTAGA +33

AACACGGAAGGA------ins 34----**ATTACCCTGTTATCCCTA**TCTAGATATGAAA +34

- **Deletion: 35/135 (26%)**

CTAGAGCAACACGGAAGGA**AT**------del 98-----------------CGAGCTCGCCCGGG -98

CTAGAGCAACACGGAAGGA**AT**------del 9---------**ATCCCTA**TCTAGATATGAAA -9

CTAGAGCAACACGGAAGGA**ATTACCCTG**-----------del 329----------- CAGAACT -329

CTAGAGCAACACGGAAGGA**ATTACCCT**------del 9---------**A**TCTAGATATGAAA -9

CTATATAAGCAGAG--------------------------del 129--------------------------ATATGAAA -129

CTAACCGCCGCCACCATGG**ATTACCC**----------del 9-----**TA**TCTAGATATGAAA -9

CTAGAGCAACACGGAAGGA**ATTA**-----------del 8--**TCCCTA**TCTAGATATGAAA -8

CTAGAGCAACACGGAAGGA**ATTACCCT**----del 9------------**A**TCTAGATATGAAA -9

CTAGAGCAACACGGAAGGA**ATTACCCT**----del 9------------**A**TCTAGATATGAAA -9

CTAGAGCAACACGGAAGGA**ATTACCCT**----del 9------------**A**TCTAGATATGAAA -9

CTAGAGC----------------------del 32----------------------------------------TAGATATGAAA -32

CTAGAgCaaCacGgaa------------del 38---------gCCATgTAGTGTATTGACCGA -38

TCAATGGGAGTTTGTTT--------------del 221--------**ATCCCTA**TCTAGATATGAAA -221

CTAGACCACCACGGAAGGA**ATTACCCTGT**-------del 6----**TA**TCTAGATATGAAA -6

TAGAGCAACACGGAAG--------------------------del 276----------CTGTCACAACTCCT -276

CTAGAGCAACACGGAAGGA**ATT**------del 10-----------**CCCTA**TCTAGATATGAAA -10

CTAGAGCAACACGGAAGGA**ATT**------del 10-----------**CCCTA**TCTAGATATGAAA -10

CTAGAG CAACACGGAAGGA----------del 112-------- GGGGATCCTCTAGAGTCG -112

CTAGAGCAACACGGAAGGA**ATTACCCT**-----del 9------------**A**TCTAGATATGAAA -9

CTAGAGCAACACGGAAGGA**ATTA**-----del 8---------**TCCCTA**TCTAGATATGAAA -8

CTAGAGCAACACGGAAGGA----------del 112-------GGGGATCCTCTAGAGTCGAG -112

CTAGAGCAACACGGAAGGA**ATTA**----------del 8-----**TCCCTA**TCTAGATATGAAA -8

CTAGAGCAACACGGAAGGA**ATTACCCTGTTATC**-------del 40----GATTCCTTGCGG -40

CTAGAGCAACACGGAAGGA**ATTACCCT**----del 2---**TATCCCTA**TCTAGATATGAAA -2

CTAGAGCAACACGGAAGGA**ATTACCCT**----------del 9-----------**A**TCTAGATATGAAA -9

CTAGAGCAACACGGAAGGA**ATTACCCTGT**-------del 9--------------CTAGATATGAAA -9

TTATCGAAATTAATAC--------------del 337---------------------TCCTAGCTGTCACTCAAG -337

CTAGAGCAACACGGAAGG**AAT**------del 8-------------**TATCCCTA**TCTAGATATGAAA -8

CTCTAGAGCAACACGG------------------del 254---------CGCTTGCTGCTGCTGCTGCT -254

TAGAGCAACACGGAAGGA**ATTACCCT**------del 9----------------**A**TCTAGATATAAAA -9

AAGGAATTACCCTGTTATCCCTA------------del 296--------GCTGGGGAAGGAAGGGGA -296

CTAGAGCAACACGGAAGGA**AT**------del 8--------------**TATCCCTA**TCTAGATATGAAA -8

CTAGAGCAACACGGAAGGA**ATTACCCT**-----del 9-----------------**A**TCTAGATATAAAA -9

CTAGAGCAACACGGAAGGA**ATTACCC**-------del 8-------------**CTA**TCTAGATATAAAA -8

CTAGAGCAACACGGAAGGA**ATTACC**-----del 3------TTATCCCTATCTAGATATGAAA -3

- **Deletion / Insertion: 14/135 (10%)**

GTACGGTGGGAGGTCTATATA---del 126/ins 2------**CCCTA**TCTAGATATGAAA -126+2

AAGCAGAGCTCTCTGGCTAA-----del 208/ins 4-----GGATCCTCTAGAGTCGAG -208+4

CAACGGGACTTTCCAAAA-------del 189/ins 7---T**ATCCCTA**TCTAGATATGAAA -189+7

TAGGGAGACCCAAGCT---------del 41/ins 21---------GTCCCTATCTAGATATGAA -41+21

CGCTCTCACGGAAGGAATTACCCTGT---del6/ins12-----TATCTAGATATGAAA -6+12

CTAGCGCTCTAGAGCA--------del 3/ins37---CGGCAGGGATTACCCTGTTATCC -3+37

CTAGAGCAACACGGAAGGA**ATTACCCTGT**------del 200/ins 10----AGCTCAAGG -200+10

ACCAAAATCAACGGGACTTT------------del 212/ins19------AAATCACGCCATGTA -212+19

CTAGAGCAACACGGAAGGA-------del 10/ins 4----**TATCCCTA**TCTAGATATGAAA -10+4

GCTTACTGGCTTATCGAAAT------------del 118/ins 34-------TCCTTGCGGTCCGAAT -118+34

CTAGAGCAACACGGAAGGA------del 10/ins 4----ATATCCCTATCTAGATATGAAA -10+4

TACCCTGTTATCCCTA-------------del 310/ins 11-----------GGGAATCAGCAGAACTG -310+11

GCACCAAAATCAACGGG--------------del 326/ins 360------------ATATACACACACCT -326+360

CAACACGGAAGGA**ATTA**-------del31/ins 10---------TCCATGTAGTGTATTGACCGA -31+10
