# Supplementary material for: 53BP1 Protects against CtIP-Dependent Capture of Ectopic Chromosomal Sequences at the Junction of Distant Double-Strand Breaks
Source: PLoS Genet. 2016 Oct 31;12(10):e1006230. doi: 10.1371/journal.pgen.1006230 (PMC5087911; doi:10.1371/journal.pgen.1006230)
Supplement: S3 Supplementary information — (DOCX) [file pgen.1006230.s003.docx]

***S3***

**Impact of CtIP *versus* 53BP1, on close and distant DSEs**

**53BP1 siRNA, Distant DNA ends (CD4-3200bp) GC92 cells**

Total number of sequences: 163

- **HiFi: 48/163 (29%)**

CTAGAGCAACACGGAAGGA**ATTACCCTGTTATCCCTA**TCTAGATATGAAA 46X

CTAGAGCAACACGGAAGGA**ATTACCCTGTT----CCCTA**TCTAGATATGAAA 1X

CTAGAGCAACACGGAAGGA**ATTACCCTGT--ATCCCTA**TCTAGATATGAAA 1X

- **Insertion : 5/163 (3%)**

CAAATGGGCGGTAGGCGTGTACGG---ins 78--- TGGGAGGTCTATATAAGCAG +78

AACACGGAAGGA**ATTACCCTGTTAT**----ins 131-----**CCCTA**TCTAGATATGAA +131

AACACGGAAGGA**ATTACCCTGTTAT**----ins 139-----**CCCTA**TCTAGATATGAA +139

AACACGGAAGGA**ATTACCCTGTTAT**----ins 140-----**CCCTA**TCTAGATATGAA +140

CGGAAGGA------------ins 145---------**ATTACCCTGTTATCCCTA**TCTAGATATGA +145

- **Deletion : 73/163 (45%)**

CTAGAGCAACACGGAAGGA**ATTACC**--------del 10--------**TA**TCTAGATATGAAA -8

CTAGAGCAACACGGAAGGA**ATTACCCTGT**--------del 9-------CTAGATATGAAA -9

CTAGAGCAACACGGAAGGA**ATTA**-------del 14-----------------TCTAGATATGAAA -14

CTAGAGCAACACGGAAGGA**A**-------------del 16---------------**A**TCTAGATATGAAA -16

CTAGAGCAACACGGAAGGA**ATTACCCT**------ del 9---------**A**TCTAGATATGAAA -9

AGGGAGACCCAAGCTGGCTA-----------del 42-------------------TCTAGATATGAAA -42

CTAGAGCAACACGGAAGGA**A**--------del 9-------**TTATCCCTA**TCTAGATATGAAA -9

CTAGAGCAACACGGAAGGA**ATTACCC**-------del 8--------**CTA**TCTAGATATGAAA -8

CTAGAGCAACACGGAAGGA**ATTA**-------del 8--------**TCCCTA**TCTAGATATGAAA -8

CTAGAGCAACACGGAAGGA**AT**-----------------del 30------CACGCCATGTAGTGTA -30

CTAGAGCAACACGGAAGGA**ATTA**-----del 8----------**TCCCTA**TCTAGATATGAAA -8

CTAGAGCAACACGGAAGGA**ATTACCCTGT**-------------del 18---------------------AAA -18

CTAGAGCAACA-----------------------del 48----------- TGTAGTGTATTGACCGATTCC -48

CTAGAGCAACACGGAAGGA**ATTA**-------del 8---------**TCCCTA**TCTAGATATGAAA -8

CTAGAGCAACACGGAAGGA**ATTA**-------del 8---------**TCCCTA**TCTAGATATGAAA -8

CTAGAGCAACACGGAAGGA**ATTA**------del 8----------**TCCCTA**TCTAGATATGAAA -8

CTAGAGCAACACGGAAGGA**ATTACCCT**-----del 5-----**CCCTA**TCTAGATATGAAA -5

CTAGAGCAACACGGAAGGA**ATTACCCT**--del 2—**TATCCCTA**TCTAGATATGAAA -2

CTAGAGCAACACGGAAGGA**ATTACCCT**-------del 9------------**A**TCTAGATATGAAA -9

ACCCACTGCTTACTGGCT ------------------------------del 86------**A**TCTAGATATGAAA -86

CTAGAGCAACACGGAAGGA**ATTACCCT**-------del 9------------**A**TCTAGATATGAAA -9

CTAGAGCAACACGGAAGGA**ATTACCCT**-------del 9------------**A**TCTAGATATGAAA -9

CTAGAGCAACACGGAAGGA**ATTACCCT**-------del 9------------**A**TCTAGATATGAAA -9

CTAGAGCAACACGGAAGGA**ATTACCCT**-------del 9------------**A**TCTAGATATGAAA -9

CTAGAGCAACAC----------------------del 177----------GGAAGGACTGGCCAGAGGCT -177

CTAGAGAACACGGAAGGA**ATTACCCT**---del 3-----**ATCCCTA**TCTAGATATGAAA -3

CTAGAGCAACACGGAAGGA**ATTACCCT**------del 9-----------**A**TCTAGATATGAAA -9

CTAGAGCAACACGGAAGGA**ATTA**-----del 8----------**TCCCTA**TCTAGATATGAAA -8

CTAGAGCAACACGGAAGGA**ATTACCCT**-----del 9-----------**A**TCTAGATATGAAA -9

TTTCCAAGTCTCCACCCC---------------------del 248-----------**TA**TCTAGATATGAAA -248

CTAGAGCAACACGGAAGGA**ATTAC**------del 11-------------**TA**TCTAGATATGAAA -11

CTAGAGCAACACGGAAG-------------del 275----------CTGTCACAACTCCTAGCTGT -275

TTAGACCACCACGGAAGGA**ATT**-------del 9----------**TCCATA**TCTAGATATGAAA -9

CTAGAGCAACACGGAAGGA**ATTACCCT**-----del 9-----------**A**TCTAGATATGAAA -9

CTAGAGCAACACGGAAGGA**ATTACCCT**--------del 9--------**A**TCTAGATATGAAA -9

CTAGAGCAACACGGAAGGA**ATTACCCT**--------del 9--------**A**TCTAGATATGAAA -9

CTAGAGCAACACG-------------del 42--------------------CCATGTAGTGTATTGACCGA -42

CTAGAGCAACACGGAAGGA**ATTACCCT**--------del 9--------**A**TCTAGATATGAAA -9

CTAGAGCAACACGGAAGGA**ATTACCCT**--------del 9--------**A**TCTAGATATGAAA -9

CTAGAGCAACACGGAAGGA**ATTACCCT**--------del 9--------**A**TCTAGATATGAAA -9

AAGCTGGCTAGCGCTCTAGAG----del 262-----------CGCTTGCTGCTGCTGCTGCTG -262

CTAGAGCAACACGGAAGGA**ATTACC**------------del 10-----**TA**TCTAGATATGAAA -10

AAAATCAACGGGACTTT----------del 193----------**TTATCCCTA**TCTAGATATGAAA -193

CTAGAGCAACACGGAAGGA-------------del2 4-------TATGAAATCACGCCATGTAG -24

CTGGCTAGCGCTCTAGAGC-----------del 322----------------GCTGGTGCTGGGGAAG -322

CTGCTTACTGGCTTATC---------------del 85--------TAGATATGAAATCACGCCATGT -85

AGCTGGCTAGCGCTC--------del 38-------------TAGATATGAAATCACGCCATGTA -38

GGAAGGAATTACCCTGT-----------------------------------del 220--------GCCGAGCCA -220

CACGGAAGGAATTACCCT---------------del 186--------------TCCCAACCAACAA -186

CGCTCTAGAGCAAACG---------------del 34-------------------AAATCACGCCATGTAG -34

CCAAAATCAACGGGACTTTCC-----------del 197-----------**CTA**TCTAGATATGAAA -197

AAAATCAACGGGACTTTCC---------------del 320----------CTATCTAGATATGAAA -320

AGAGCAACACGGAA---------------del 162--------------GCAGAGTGAAGGAAGGA -162

AGAGCTCTCTGGCTAAC---------------del 128------------GCCATGTAGTGTATTGACC -128

CTAGCGCTCTAGAGCAACACG----------del 21-------------CTATCTAGATATGAAAT -21

CACGGGGATTTCCAAGTCT-------------------del 259------------AGATATGAAATCACG -259

CTAGAGCAACACGGAAGGA**ATTACCCT**----------del 9-------**A**TCTAGATATGAAA -9

CTAGAGCAACACGGAAGGA**ATTACCCT**----------del 9-------**A**TCTAGATATGAAA -9

CCCAGCTGGCTAGCGCTCTAGAGC------------del 32------------------TAGATATGAAA -32

CTAGAGCAACACGGAAGGA**ATTACCC**----del 8------------**CTA**TCTAGATATGAAA -8

CTAGAGCAACACGGAAG-------------del 30---------AAATCACGCCATGTAGTGTATT -30

CTAGAGCAACACGGAAGGA**ATTACCCT**-----del 9--------------**A**TCTAGATATGAAA -9

CTAGAGCAACACGGAAGGA**ATTACCCT**-----del 9--------------**A**TCTAGATATGAAA -9

CTAGAGCAACACGGAAGGA**ATTACCCT**-----del 9--------------**A**TCTAGATATGAAA -9

CTAGCGC TCTAGAGC---------------del 49--------------GCCATGTAGTGTATTGACCGA -49

CTAGAGCAACACGGAAGGA**ATTACC**-----del 10-------------**TA**TCTAGATATGAAA -10

CTAGAGCAACACGGAAGGAA----------del 25-------AATCACGCCATGTAGTGTATTG -25

CTAGAGCAACACGGAAGGA**ATTACCCT**-----del 9--------------**A**TCTAGATATGAAA -9

CTAGAGCAACACGGAAGGA**ATTACCCT**-----del 9--------------**A**TCTAGATATGAAA -9

CTAGAGCAACACGGAAGGA**ATTACCCT**------del 8-------------**A**TCTAGATATGAAA -8

CTGCATAGCAACACGGAACGA**ATTAC**----del 8-------------**CCCTA**TCTAGATATGAA -8

TATAGAGCAACCCGGAAGGA**ATTA**--------del--9 ----**CCCTA**TCTAGATATGAAATCA -9

CTCTAGAGCAACACGGAA----------------del 213---------------GCTCAAGGAGACCACCA -213

- **Deletion / Insertion : 37/163 (23%)**

TGGTGATGCGGTTTTGGC--------------------del 712/ins 328------------------------GC -712+328

CTAGAGCAACACGGAAGGA**AT**--------del 12/ins 4----**CCTA**TCTAGATATGAAA -12+4

CGGTTTTGGCAGTACATCAAT-----del 62/ins 24-------- **CCTA**TCTAGATATGAA -62+24

CTAGAGCAACACGGAAGGA**A**---------del 241/ins 283----CTCTTAGGCGCTTG -241+283

CTAGAGCAACAC--------------del 428/ins 17-----------------------------------------------C -428+17

CTAGAGCAACACGGAA----------del 19/ins 2-----------------**TA**TCTAGATATGAAA -19+2

GGTGATGCGGTTTTGGC-----------------del 713/ins 380----------------------------C -713+380

---------------------------------------del 331/ins84-------------GTAGTGTATTGACCGATT -331+84

---------------------------------del 318/ins 189--- **TGTTATCCCTA**TCTAGATATGAAA -318+189

---------------------------------del 322/ins 190--------------**TCCCTA**TCTAGATATGAAA -322+190

GGGCGTGGATAGCGGT------del 56/ins 95------ TAGATATGAAATCACGCCATG -62+59

CTAGAGCAA----------del 21/ins 95-------- **ATCCCTA**TCTAGATATGAAATCACG -21+95

CTAGAGCAACACGGAAGGA------del 13/ins 2-------**CCCTA**TCTAGATATGAAA -13+2

AGTGAAGGAAGGACT-------------del 243/ins 166---------------------------------------- -243+166

CTAGAGCAACACGGAAGGA**ATTACCCT**------del 136/ins 135-----GTCACAACT -136+135

CCATTGACGTCAATG-----------del 233/ins 308--------------**TCCCTA**TCTAGATAT -233+308

attAaTaCGaCTCaCTATAG---------del 62/ins 3----------**TAGATA**TGAAATCAC -62+3

CTAGAGCAACACGGAA-----del 25/ins 3-----------------------------------GATATGAAA -25+3

TGGTGATGCGGTTTTGGC---------------del 712/ins204----------------------------------GC -712+204

CTAGCGCTCTAGAGCAACACGGA----del 18/ins 6--------CCTATCTAGATATGAAA -18+6

TGGT-------------------del 314/ins 322---- TGTTATCCCTATCTAGATATGAAATCA -314+322

ACTTTCCAAAATGTCGTAACA----del 178/ins 6---TATCCCTATCTAGATATGAAA -178+6

AGAGCCACACGGAAGGAATT----------del26/ins 8---ATCACGCCATGTAGTGTA -26+8

CCAAAATGTCGTAACA------------del 178/ins 6--------TATCCCTATCTAGATATG -178+6

CTGGCTAGCGCTCTAGAGCAACA-G----del 278/ins 19-----GCAGCTGTCACAAC -278+19

CTAGAGCAACACGGAAGG-------------------------del 369 ins 253------------------ -369+253

CTAGAGCAACACGGAAGGA**A**------del 10/ins 2------**ATCCCTA**TCTAGATATGAA -10+2

TTCCAAAATGTCGTAACA-----------del 179/ins 290----------ATTACCCTGTTATC -179+290

AATGGGCGGTAGG ---------------del 154/ins 143--CTATCTAGATATGAAATCACG -154+143

GACCGATTCCTTGCGGTCCGAATGGGCCGAACCC------del 340/ins 311----------- -340+311

CGCTCTAGAGCAACACGGAAGG--------del 19/ins 214---------------TCTAGATAT -19+214

CATTGACGTCAATGGGA---------del 107/ins 18---ACACACACCTGTGCAAGAAGC -107+18

GACTCACTATAGGGAGACCCAAG----del 53/ins 2-----ATGAAATCACGCCATGTA -53+2

TGGGAGTTTGTTTTGGCACCAA----------del 235/ins 307-------GCCATGTAGTGTAT -235+307

CTAGAGCAACACGGAAGGA---------del 10/ins 5----TATCCCTATCTAGATATGAA- -10+5

CTAGAGCAACACGGAAGGA**ATTACCC**-----del 4/ins 4--**ATCCCTA**TCTAGATATG -4+4

CAGAATCGGGAGACCCAA-----------del 46/ins 12---TCCCTATCTAGATATGAAA -46+12

**CtIP siRNA, Distant DNA ends (CD4-3200bp) GC92 cells**

Total number of sequences: 165

- **HiFi: 94/165 (57%)**

CTAGAGCAACACGGAAGGA**ATTACCCTGTTATCCCTA**TCTAGATATGAAA 87X

CTAGAGCAACACGGAAGGA**ATTACCCTGT**----**CCCTA**TCTAGATATGAAA

CTAGAGCAACACGGAAGGA**ATTACCCTG--**----**CCCTA**TCTAGATATGAAA

CTAGAGCAACACGGAAGGA**ATTACCCTGTT**--**CCCTA**TCTAGATATGAAAT 2X

CTAGAGCAACACGGAAGGA**ATTACCCTGT-ATCCCTA**TCTAGATATGAAA 2X

CTAGAGCAACACGGAAGGA**ATTACCCTGT-----CCCTA**TCTAGATATGAAA 1X

- **Insertion : 4/165 (2%)**

TAGAGCAACACGGAAGGA**ATTACCCTGTTTATCCCTA**TCTAGATATGAAAT +1

TAGAGCAACACGGAAGGA**ATTACCCTGT**---ins 14----**TATCCCTA**TCTAGAT +14

TAGAGCAACACGGAAGGA---ins 147----**ATTACCCTGTTATCCCTA**TCTAGAT +147

TAGAGCAACACGGAAGGA**ATTACCCTGTTAT**----ins 5-**CCCTA**TCTAGATAT +5

- **Deletion : 52/165 (32%)**

CTAGAGCAACACGGAAGGA**ATTACCCTGTT** ---del 6--- **A**TCTAGATATGAAA -6

CTAGAGCAACACGGAAGGA**ATTACCC**----del 8----**CTA**TCTAGATATGAAA -8

CTAGAGCAACACGGAAGGA**ATTACCCT**------del 9---**A**TCTAGATATGAAA -9

CTAGAGCAACACGGAAGGA**ATTACCCT**----del 9-----**A**TCTAGATATGAAA -9

CTAGAGCAACACGGAAGGA**ATTAT**---del 8-----**CCCTA**TCTAGATATGAAA -8

CTAGAGCAACACGGAAGGA**ATTAT**-----del 8---**CCCTA**TCTAGATATGAAA -8

CTAGAGCAACACGGAAGGA**ATTAT**-----del 8---**CCCTA**TCTAGATATGAAA -8

CTAGAGCAACACGGAAGGA**ATTAT**----del 8----**CCCTA**TCTAGATATGAAA -8

CTAGAGCAACACGGAAGGA**ATTAT**----del 8----**CCCTA**TCTAGATATGAAA -8

CTAGAGCAACACGGAAGGA**ATTACCCTGTTA**----del 10------GATATGAAA -10

CTAGAGCAACACGGAAGGA**ATTACCCTG**---del 9------**T**CTAGATATGAAA -9

CTAGAGCAACACGGAAGGA**ATT**-------del 12----------**CTA**TCTAGATATGAAA -12

CTAGAGCAACACGGAAGGA**ATTACCCTGT**---------del 13---------ATATGAAA -13

CTAGAGCAACACGGAAGGA**ATA** -------------del 23------------------------TGAAA -23

CTAGAGCAACACGGAAGGA-----------del 24-----------------------------TATGAAA -24

CAAGCTGGCTAG--------------------------del 43---------------------TAGATATGAAA -43

CTAGAGCAACACGGAAGGA**ATTACCCTGT**----del 9----CTAGATATGAAAT -9

CTAGAGCAACACGGAAGGA**ATTA**---del 8----**TCCCTA**TCTAGATATGAAAT -8

CTAGAGCAACACGGAAGGA**ATTACCCT**-----del 9----**A**TCTAGATATGAAAT -9

--------------------------------------------------------del 236------------------------------------ -236

---------------------------------------------del 184-------------------------------------------------- -184

----------------------------------------del 57------------------------------------------------------- -57

----------------------------------------del 339------------------------------------------------------- -339

-----------------------------------------del 311------------------------------------------------------ -311

CTAGAGCAACACGGAAGGA**ATTACCCT**------del 9----**A**TCTAGATATGAAAT -9

CTAGAGCAACACG--------------------------del 29-------------------GATATGAAAT -29

ACGGAAGGA**ATTACCCTGTTATCC**------------del 1------------**TA**TCTAGATAT -1

CTAGAGCAACACG------------------------del 28-----------------------GATATGAAAT -28

CTAGAGCAACACGGAAGGA**ATTA**----del 8--**TCCCTA**TCTAGATATGAAAT -8

CTAGAGCAACACGGAAGGA**ATTAC**--------------del 30----------------------------- -30

CTAGAGCAACACGGAAGGA**ATTACCCTGTTAT**---del 4-**A**TCTAGATATGAAA -4

CTAGAGCAACACGGAAGGA**AT**-----del 8---**TATCCCTA**TCTAGATATGAAA -8

CTAGAGCAACACGGAAGGA**ATTACCCT**-----del 9-----**A**TCTAGATATGAAA -9

CACGGAAGGA**ATTACCCT**------del 9---ATCTAGATATGAAATCACGCCATG -9

TTTCCAAAATGTCGTAACAA-------del 180-----TATCTAGATATGAAATCACG -180

CACGGAAGGA**ATTACCCT**-----del 9----**A**TCTAGATATGAAATCACGCCATGT -9

CTAGAGCAACACGGAAG--------del 25---------ATATGAAATCACGCCATGTAGT -25

CTAGAGCAACACGGAAGGA**AT**-----del 8----**TATCCCTA**TCTAGATATGAAAT -8

GCAACACGGAAGGAATTACC--------------del 232-----------CTCTCTTAGGCGCTT -232

CTAGCGCTCTAGAGCAACACGGAAGGA----------del 209-------GCTCAAGGAGA -209

CTAGAGCAACACGGAAGGA**ATTACCCT**-------del 9-----------**A**TCTAGATATGAAAT -9

CTAGAGCAACACGGAAGGA**ATT**-------del 10-----------**CCCTA**TCTAGATATGAAAT -10

CTAGAGCAACACGGAAGGA**ATTACCCTG**-----del 8----------**A**TCTAGATATGAAAT -8

CTAGAGCAACACGGAAGGA**ATTACCCT**---del 5------**CCCTA**TCTAGATATGAAAT -5

CTAGAGCAACACGGAAGGA**ATTACCCT**--------del 9----------ATCTAGATATGAAAT -9

CTAGAGCAACACGGAAGGA**ATTACCCT**--------del 9---------ATCTAGATATGAAAT -9

CTAGAGCAACACGGAAGGA**ATTACC**---------------del 231-------------ATCTCTCTTAG -231

CTAGCGCTCTAGAGCAACA-----------del 18----------**TATCCCTA**TCTAGATATGAAA -18

CTAGAGCAACACGGAAGG-------del 11--------------**TATCCCTA**TCTAGATATGAAA -11

CAAAATGTCGTAACAAC---------------del 176--------TATCCCTATCTAGATATGAAA -176

GGAGACCCAAGCTGGC----------------del 45------------TAGATATGAAATCACGCCAT -45

CTAGCGCTCTAGAGCAAC---------del 28--------TAGATATGAAATCACGCCATGTAG -28

- **Deletion / Insertion : 15/165 (9%)**

CAACACGGAAGGA**ATTA**---del7/ins---**ATCCCTA**TCTAGATATGAAA -7+5

GGAAGGA---------------------------del 104/ins 45------------------------TAGAGT -104+45

CTAGAGCAACACGGAA----------del 19/ ins 2-----**TA**TCTAGATATGAAA -19+2

ACACGGAAGGAATTACCCT------del 223/ins 1-----CCGAGCCATCTCT -223+1

CTAGAG-----------------------del 27/ins 79-------CCTATCTAGATATGAAAT -27+79

GCCCATCGAAATTAATA-------------del 73/ins 22---AGATATGAAATCACGC -73+22

TAGAGCAACACGGAAGGA**ATTACCC**---del2/ins 1--**TATCCCTA**TCTAGATAT -2+1

CCACCCCATTGACGTCAATGG--------del 233/ins 12--**CCCTA**TCTAGATATGAA -233+12

TAGAGCAACACGGAAGGAAT-------del 11/ins 3---ACCCCTATCTAGATATGAAAT -11+3

GTAAAGAGCAACCCGGAAGGA-----del 16/ins 2------**TA**TCTAGATATGAAATCAC -16+2

TAATACGACTCACTATAGGGAGACCCAAGCTG-----del 406/ins 181--------------- -406+181

TAGAGCAACACGGAAGGA**ATT**------del 6/ins 6----**TTATCCCTA**TCTAGATATGA -6+6

CAACACGGAAGGA**ATTACCCT**-----del 1/ins138--**TTATCCCTA**TCTAGATATGA -1+138

CAACACGGAAGGA**ATTACCCTGT**----del 4/ins 10---**CCTATCTA**GATATGAAAT -4+10

CTAGAGAACCCACTGCTTACTGG---- del 83/ins 1--**TCCCTA**TCTAGATATGAAA -83+1

**53BP1+CtIP siRNA, Distant DNA ends (CD4-3200bp) GC92 cells**

Total number of sequences: 95

- **HiFi: 40/95 (42%)**

CTAGAGCAACACGGAAGGA**ATTACCCTGTTATCCCTA**TCTAGATATGAAA 35X

CTAGAGCAACACGGAAGGA**ATTACCCTGTT---CCCTA**TCTAGATATGAAA 2X

CTAGAGCAACACGGAAGGA**ATTACCCTG**----**ATCCCTA**TCTAGATATGAAAT 2X

CTAGAGCAACACGGAAGGA**ATTACCCTGT------CCCTA**TCTAGATATGAAA 1X

| - **Insertion : 0/95 (0%)** |  |
| --- | --- |

- **Deletion : 42/95 (44%)**

| CTAGAGCAACACGGAAGG**AATTACCC**---del 6----**CCCTA**TCTAGATATGAAAT  CTAGAGCAACACGGAAGG**AATTACCCT**-----del 9--------**A**TCTAGATATGAAAT  ----------------------------------------------del 24-----------------**TA**TCTAGATATGAAAT  CTAGAGCAACACGGAAG----------------del 17---------**CTA**TCTAGATATGAAAT  CTAGAGCAACACGGAAGGAAT-------del 13----------**CTA**TCTAGATATGAAAT  CTAGAGCAACACGGAAGGA**ATTACCCT**—del 9-----**A**TCTAGATATGAAAT  CTAGCGCTCTA------------------------del 34---------------------TCTAGATATGAAAT  ------------------------------------------del 359--------------------------------------------------  ------------------------------------------del 200------------------------TAGATATGAAAT  CTAGAGCAACACGGAAGGA**ATTACCCT**---del 9--------ATCTAGATATGAAAT  CTAGAGCAACACGGAAGGA**ATTACCCT**----del 9-------ATCTAGATATGAAAT  CTA-------------------------------------del 38----------------------------GATATGAAAT  CTAGAGCAACACGGAAGGA**ATTA**----del 8----**TCCCTA**TCTAGATATGAAAT  CTAGAGCAACACGGAAGGAAT-------del 12---------**CCCTA**TCTAGATATGAAAT  TAGAGCAACACGGAAGGA**ATTACCCTGT**----------del 9-------CTAGATATGAAAT  CTAGAGCAACACGGAAGGA**ATTACCCT**----------del 9------ATCTAGATATGAAAT  CTAGAGCAACA---------------------------------del 81-----------------------------------------------  CTAGCGCTCTAGAGC-----------------------del 47-------------------------------------------------  CTAGAGCAACACGGAAG-----------------------del 22------------------TAGATATGAAAT  CTAGAGCAACACGGAAGGA**A**-----------del 8------**TTATCCCTA**TCTAGATATGAAAT  CTAGAGCAACACGGAAGGA**AT** ---------del 9--------**ATCCCTA**TCTAGATATGAAAT  CTAGAGCAACACGGAAGGAAT--------del 8----------TATCCCTATCTAGATATGAAA  CTAGCGCTCTAGAGCAACACGGAA--------del 15-------TCCCTATCTAGATATGAAA  CTAGAGCAACACGGAAGGAATTAC---del 5--------TATCCCTATCTAGATATGAAAT  CTAGCGCTCTAGAGCAACACGGAAGGAAT---del 8-TATCCCTATCTAGATATGAAA  CTAGAGCAACACGGAAGGA**AT**---------del 15--------------**A**TCTAGATATGAAATCAC  CTAGAGCAACACGGAAGGA**AT**-----del 8--------**TATCCCTA**TCTAGATATGAAATCA  CTAGAGCAACACGGAAG--------------del 65-----CGGTCCGAATGGGCCGAACCCGGT  CTAGAGCAACACGGAAGGA**ATT**---------del 10-----------**CCCTA**TCTAGATATGAAA  CTATAGGGAGACCCAAGCTGGCT----------del 214---------CAGATTCCCAACCAACAA  CTAGAGCAACACGGAAGGA**ATTACCCT**-------del 9-----------**A**TCTAGATATGAAAT  AGAGCAACACGGA-------------------del 303--------------AGGGAAGACGCTGGTGCTGG  CAAGTCTCCACCCCATTGACG-------------del 262---------CCATGTAGTGTATTGACCG  TTCCAAGTCTCCACCCCATTGACGTC------del240-----------TATCTAGATATGAAATC  AACACGGAAGGA**ATTACCCTGTTATCC**-del 1---**TA**TCTAGATATGAAATCACGCC  AGCGCTCTAGAGCAACACGGAAGGA**AT**--del 8-**TATCCCTA**TCTAGATATGAAA  CTAGAGCAACACGGAAGGA**ATTACCCTGT**--------del 9------**CTA**GATATGAAATC  CCAAAATGTCGTAACAACT------------de182------------ATCTAGATATGAAATCACG  GCTAGCGCTATAGAGCAA-----------del 42--------------CACGCCATGTAGTGTATTGAC  ACACGGAAGGAATTACCCTGT------del 11-----AGATATGAAATCACGCCATGTAG  TCTAGAGCAACACGGAAGGAAT-----del 8---------TATCCCTATCTAGATATGAAA  CTAGAGCAACACGGAAGGA**ATTACCCT**----del 9-----ATCTAGATATGAAATCACGC   - **Deletion / Insertion : 13/95 (14%)**   CTAGAGCAACACGGAAGGA**ATTAC**--------del 11/ins 1----**TA**TCTAGATATGAAAT  CTAGAGCAACACGGAAGGA**ATTACCCT------**del 24/ins 21-------------------------------  CTAGA----------- del 30/ins6-------------------------------------**TA**TCTAGATATGAAAT  CTAGAGCAACACGGAAGG-----------------------del 37/ins 27 --------------------------  CTAGAGCAACAC------------------del 23/ins 30----------------TATCTAGATATGAAAT  ---------------------------------------------del 68/ins 5----------------------------------------AAAT  GACCCAAGCTGGCTAGCGCTC------del 73/ins 78-----TTCCTTGCGGTCCGAATGGG  AGTGTATTGACCGATTCCTTGCGGTC--------------------del 343/ins 323--------------------  CTAGAGCAACACGGAAGGAATTACCCTGT---del 2/ins 3-TCCCTATCTAGATATGAA  GACCCAAGCTGGCTAGCGCTC-----del 73/ins 82----TTCCTTGCGGTCCGAATGGGCC  CTAGCGCTCTAGAGCAACACGGAA-----del 13/ins 8---TATCCCTATCTAGATATGAA  GACCCAAGCTGGCTAGCGCTCTAGA-------del 274/ins 35--------GCTGCTGCTGCAGC  GCGCTCTAGAGCAACACGGAAG-----------del 289/ins 1------------------AGCTGTCACT | -6  -9  -24  `-17  -13  -9  -34  -359  -200  -9  -9  -38  -8  -12  -9  -9  -81  -47  -22  -8  -9  -8  -15  -5  -8  -15  -8  -65  -10  -214  -9  -303  -262  -240  -1  -8  -9  -182  -42  -11  -8  -9  -11+1  -24+21  -30+6  -37+27  -23+30  -68+5  -73+78  -343+323  -2+3  -73+82  -13+8  -274+35  -289+1 |
| --- | --- |

**53BP1 siRNA, Distant DNA ends (CD4-3200bp) GC49 cells**

Total number of sequences: 114

- **HiFi: 21/114 (18%)**

CTAGAGCAACACGGAAGGA**ATTACCCTGTTATCCCTA**TCTAGATATGAAA 21X

- **Insertion : 3/114 (3%)**

AGAGCAACACGGAAGGA**ATTACCCTGT**-------ins 219-------**TATCCCTA**TCTAG +219

AGCAACACGGAAGGA----------ins 73------**ATTACCCTGTTATCCCTA**TCTAG +73

AGCAACACGGAAGGA**A**----ins 21------------**TTACCCTGTTATCCCTA**TCTAG +21

- **Deletion : 71/114 (62%)**

CTAGCGCTCTAGAGCAACACGGAAGGA--------del 24-----------TATGAAATCAC -24

CTAGCGCTCTAGAGCAACAC-------------del 21--------CCTATCTAGATATGAAAT -21

CTAGAGCAACACGGAAGGA--------------del 24------------TATGAAATCACGCCA -24

ACGGAAGGAATTACCCTGT-------------del 77------------CACCCATTCGAATTCGAG -77

AGAGCAACACGGAACGA**A**----del 8---------**TTATCCCTA**TCTAGATATGAAATCA -8

GGAGACCCAAGCTGGCTA----------del 42---------------TCTAGATATGAAATCACG -42

CTAGAGCAACACGGAAGGA**ATTACCCT**----del 9-----**A**TCTAGATATGAAATCACG -9

CTAGAGCAACACGGAAGGA------------del 24------------TATGAAATCACGCCATG -24

CTAGCGCTCTAGAGCAACAC-------------del 21--------CCTATCTAGATATGAAATC -21

TAGAGCAACACGGAAGGA**ATTACCCT**------del 9------------**A**TCTAGATATGAAATC -9

ATAGGGAGACCCAAGCTGGCTA------------del 42--------------TCTAGATATGAAATCA -42

CTAGAGCAACACGGAAGGA**ATTACCCT**-----del 9-----------**A**TCTAGATATGAAA -9

CTAGAGCAACACGGAAGGA**ATTACCCT**-----del 9-----------**A**TCTAGATATGAAA -9

CTAGAGCAACACGGAAGGA**ATTACCCT**-----del 9-----------**A**TCTAGATATGAAA -9

CTAGAGCAACACGGAAGGA**AT**--------del 8-------**TATCCCTA**TCTAGATATGAAA -8

GGCTTATAGAAATTAATACGACTC-----del 63---------CCTATCTAGATATGAAATCA -63

CTAGAGCAACACGGAAGGA**ATTACCCT**-----del 9-----------**A**TCTAGATATGAAA -9

CTAGAGCAACACGGAAGGA**ATTACCCT**-----del 9-----------**A**TCTAGATATGAAA -9

CTAGAGCAACACGGAAGGA**ATTACCCT**-----del 9-----------**A**TCTAGATATGAAA -9

CTAGAGCAACACGGAAGGAATTACCC-----------del 191---------AACCAACAAGA -191

CTAGAGCAACACGGAAGGA**ATTACCCTGTTATC**---del 6---TAGATATGAAATC -6

CTAGAGCAACACGGAAGGA**AT**-----del 8---------**TATCCCTA**TCTAGATATGAAA -8

CTAGAGCAACACGGAAGGA**ATTACCCT**------del 9---**A**TCTAGATATGAAATCAC -9

CTAGAGCAACACGGAA----------del 13-------**TATCCCTA**TCTAGATATGAAATCAC -13

CTAGAGCAACACGGAAGGA**A**------------del 18-----------------ATCACGCCATGTAGT -18

CTAGAGCAACACGGAAGGA**ATTACCCT**------del 9---**A**TCTAGATATGAAATCAC -9

CTAGAGCAACACGGAAGGA**ATTACCCTG**---del 6---**CTA**TCTAGATATGAAATCA -6

CTAGAGCAACACGGAAGGA**ATTACCCTGTTAT**-del 1-**CCTA**TCTAGATATGAA -1

CTAGAGCAACACGGAAGGA**ATTACCCT**------del 9---**A**TCTAGATATGAAATCAC -9

CTAGAGCAACACGGAAGGA**ATTACCCT**------del 9---**A**TCTAGATATGAAATCAC -9

CTAGAGCAACACGGAAGGA**AT**--------del 8--------**TATCCCTA**TCTAGATATGAAA -8

GCTCTAGAGCAACACG-----------del 56 --------GACCGATTCCTTGCGGTCCGAATG -56

CTAGAGCAACACGGAAGGA**ATTACCCT**---------del 127-----CATATACACACACCT -127

CTAGAGCAACACGGAAGGA**AT**------del 8----------**TATCCCTA**TCTAGATATGAAA -8

CTAGAGCAACACGGAAGGA**ATTACCCT**-------del 9-----------**A**TCTAGATATGAAA -9

CTAGAGCAACACGGAAGGA**ATTACCCTGTTATC**------del 6-----TAGATATGAAA -6

CTCTAGAGCAACACGGAA----------------------del 101--------TTCGAGCTCGCCCGGGG -101

CTAGAGCAACACGGAAGGA**ATTACCCT**-------del 9---------**A**TCTAGATATGAAAT -9

CTAGAGCAACACGGAAGGA**ATTAC**----------del 30---------------GCCATGTAGTGTAT -30

AAGCTGGCTAGCGCTC----------------del 37------TAGATATGAAATCACCCATGTAG -37

CTAGAGCAACACGGAAGGA**ATTACC**--------------del 78----GGTCACCCATTCGAAT -78

CTAGAGCAACACGGAAGGA**AT**-----del 8---**TATCCCT**ATCTAGATATGAAATC -8

CTAGAGCAACACGGAAGGA**ATTACCCT**----del 9------ATCTAGATATGAAATC -9

CTAGAGCAACACGGAAGGA**ATTACCCT**----del 9------ATCTAGATATGAAATC -9

CTAGAGCAACACGGAAGGA---------------del 30------------ATCACGCCATGTAGTG -30

CTCTAGAGCAACACGG-----------del 254----------CGCTTGCTGCTGCTGCTGCTGCA -254

CTAGAGCAACACGGAAGGA**ATTACCCT**----del 9------ATCTAGATATGAAATC -9

CTAGAGCAACACGGAAGGA**AT**------del 8---**TATCCCTA**TCTAGATATGAAATCA -8

TCTGGCTAACTAGAGAACCCAC----------del 115 -------GCCATGTAGTGTATTGACC -115

CTAGAGCAACACGGAAGGA**ATTACCCT**-----del 9--------**A**TCTAGATATGAAAT -9

CTAGAGCAACACGGAAGGA**ATTACCCT**-----del 9--------**A**TCTAGATATGAAAT -9

CTAGGGAAGCAGGGAAGGA**A**-------del 9---**TATCCCTA**TCTAGATATGAAATCA -9

CTAGAGCAACACGGAAGGA**ATTACCCT**-----del 9--------**A**TCTAGATATGAAAT -9

AAAATGTCGTAACAA-----del 174--------**TGTTATCCCTA**TCTAGATATGAAATCA -174

CTAGAGCAACACGGAAGGA**AT**------del 8---**TATCCCTA**TCTAGATATGAAATCA -8

GATTTCCAAGTCTCCACCCCATTG---------------del 382------------CAAGAAGCAGAG -382

CTAGAGCAACACGGAAGGAATTAC----del 172-----------TGGCCAGAGGCTCAGATT -172

CAAGGACAGCAGGGACGGA**ATT**------del 9-----**TCCCT**ATCTAGATATGAAATCAC -9

CTAGAGCAACACGGAAGGA-------------del 24-----------TATGAAATCACGCCATGTA -24

GGTAGGCGTGTACGGTGGGAGGTC-------del 177----------CTTGCGGTCCGAATGGG -177

CGTGTACGGTGGGAGGTCTATATAA---del 210 ------------TTCGAGCTCGCCCGGG -210

AGAGCAACACGGAACGA**ATT**---del 9------**TCCCTA**TCTAGATATGAAATCACGC -9

CTAGAGCAACACGGAAGGA**ATTACCCT**-----del 9--------**A**TCTAGATATGAAAT -9

CTAGAGCAACACGGAAGGA**ATTACCCTGT**--------del 268 ------------------CACAAC -268

CTAGAGCAACACGGAAGGA**ATTACCCT**-----del 9--------**A**TCTAGATATGAAAT -9

CTAGAGCAACACGGAAGGA**ATTAC**---------------del 172 ----------------TGGCCAGAG -172

CTAGAGCAACACGGAAGGA**ATTACCCT**-----del 9--------**A**TCTAGATATGAAAT -9

CGCTCTAGAGCAACACG-----------------del 325------------------CTGGTGCTGGGGAA -325

CTAGAGCAACACGGAAGGA**ATTACCCT**--del 5---**CCCTA**TCTAGATATGAAATC -5

GGACTTTCCAAAATGTCGTAACA------------------del 230--------------TGTAGTGTATT -230

CTAGAGCAACACGGAAGGA--------del 24-------------TATGAAATCACGCCATGTAG -24

- **Deletion / Insertion : 19/114 (17%)**

CTAGAGCAACACGGAAGGAATTA----del 11/ins 1-----CTATCTAGATATGAAA -11+1

CTATAGAGCAACACGG-----------del 19/ins 4---------CCTATCTAGATATGAAA -19+4

CGGCTAGCGCTCATGAGCAACA---del 48/ins 14-TAGTGTATTGACCGATTCC -48+14

CTAGCGCTCTAGAGCAACACGG------del 17/ins 2---TCCCTATCTAGATATGAAAT -17+2

CTAGAGCAACACGGAAGGA**ATTAC**-----del 7/ins 5----**TCCCTA**TCTAGATATGAA -7+5

CTAGAGCAACACGGAAGGA**ATTACCCT**--del 5/ins 114-**CCCTA**TCTAGATATGAA -5+114

CTAGAGCAACACGGAAGGA**ATTACCCTGTT**------del 198/ins1--GCTCAAGGAGA -198+1

GGCTAGCGCTCTAGAGC--------del 50/ins3--------------------ATGTAGTGTATTGACCG -50+3

CTAGCGCTGTAGAGCAAC-C----------del 44 /ins 3-----GCCATGTAGTGTATTGACCG -44+3

TAGAGAGACCCAA---------del 50/ins 12-----------TCTAGATATGAAATCACGCCATGT -50+12

CTAGAGCAACACGGAAGGA**A**-------del 267/ins 1-----------------TGCAGCTGTCACAA -267+1

CTAGAGCAACACGG------------del 360/ins24-------CTGCGAGAGTTCCCAGAAG -360+24

GCAGAGCTCTCTGGCTA----------del 358/ins 6--------GCTGCTGCAGCTGTCACAA -358+6

TTTCCAAG TCTC---del 238/ins448--**ATTACCCTGTTATCCCTA**TCTAGATATGAA -238+448

GCTAGCGCTCTAGAGCAACACG---------del 268/ins2-------------TGCTGCTGCAGCTG -268+2

CTAGCGCTCTAGAGCAACACGG------del 369/ins19------------TCCCAGAAGAAGATC -369+19

CAATGGGCGTGGATA----------del 279/ins269----------**TATCCCTA**TCTAGATATGA -279+269

CTAGAGCAACACGGAAGGA**ATTACCCTGTTAT**----del 51/ins45----CTAGATATGA -51+45

CTAGAGCAACACGGAAGGA**ATTACCCTGTTAT**------del 88/ins23------TCCTTGCG -88+23

**53BP1 siRNA, Close DNA ends (CD4-34bp) GCK20 cells**

Total number of sequences: 120

- **HiFi: 63/120 (52.5%)**

CTAGAGCAACACGGAAGGA**ATTACCCTGTTATCCCTA**TCTAGATATGAAA 58X

CTAGAGCAACACGGAAGGA**ATTACCCTGTT**-----**CCCTA**TCTAGATATGAAA 1X

CTAGAGCAACACGGAAGGA**ATTACCCTGT**---**ATCCCTA**TCTAGATATGAAA 4X

- **Insertion : 5/120 (4%)**

CACGGAAGGA**ATTACCCTGT**---ins 2----**TATCCCTA**TCTAGATATGAAATC +2

CACGGAAGGA**ATTACCCTGTTAT**----ins 5---**CCCTA**TCTAGATATGAAATC +5

CACGGAAGGA-----------ins 21-------------**ATTACCCTGTTATCCCTA**TCTAGA +21

CACGGAAGGA**ATTACCCTGT**-----ins 16--**TATCCCTA**TCTAGATATGAAATC +16

CACGGAAGGA**ATTACCCTGTTAT**---ins 5------**CCCTA**TCTAGATATGAAATC +5

- **Deletion: 37/120 (31%)**

CTAGAGCAACACGGAAGGA**ATTACCCT**------del 9-----------**A**TCTAGATATGAAA -9

CTAGAGCAACACGGAAGGA**AT**------del 8----------**TATCCCTA**TCTAGATATGAAA -8

CTAGAGCAACACGGAAGGA**AT**------del 8----------**TATCCCTA**TCTAGATATGAAA -8

CTAGAGCAACACGGAAGGA**ATTACCCT**--------del 9----------**A**TCTAGATATGAAA -9

CTAGAGCAACACGGAAGGA**ATTACCCT**-----del 9-------------**A**TCTAGATATGAAA -9

TCCCTAACCGCCGCCACCATGG------del 9------**TTATCCCTA**TCTAGATATGAAA -9

CTAGAGCAACACGGAAGGA**ATTACCC**--------del 53------------TTGCGGTCCGAAT -53

CTAGAGCAACACGGAAGGA**ATTACCCT**------del 9------------**A**TCTAGATATGAAA -9

CTAGAGCAACACGGAAGGA**ATTACCCT**------del 9------------**A**TCTAGATATGAAA -9

CTAGAGCAACACGGAAGGA**ATT**---------del 9---------------**TCCCTA**TCTAGATATGA -9

CTAGAGCAAC-------------------del 93---------GGTCACCCATTCGAATTCGAGCTCGC -93

CTAGAGCAACACGGAAGGA**ATTACC**-------del 10--------------**TA**TCTAGATATGAAA -10

CTAGAGCAACACGGAAGGA-----------del 112-----------GGGGATCCTCTAGAGTCGA -112

CTAGAGCAACACG--------------del 42----------------------CCATGTAGTGTATTGACCGA -42

CTAGAGCAACACGGAAGGA**AT**--------del 8-----------**TATCCCTA**TCTAGATATAAAA -8

CTAGAGCAACACGGAAGGA-----------del 112-----------GGGGATCCTCTAGAGTCGA -112

CTAGAGCAACACGGAAGGA**ATTACCCTGTTA**-------del 22----CGCCATGTAGTGTA -22

CTAGAGCAA ------------------------------------del 376-----------------GAAGATCACAGTCT -376

CTAGAGCAACCCGGAAGGA**ATTA**---del 1---**CCTGTTATCCCTA**TCTAGATATGAAA -1

CTAGAGCAACACGGAAGGA-----------del 112-----------GGGGATCCTCTAGAGTCGA -112

CTAGAGCAACCCGGAAGGA**ATTA**---del 1---**CCTGTTATCCCTA**TCTAGATATGAAA -1

CTAGAGCAACACGGAAGGA**ATTACCCTGT**---------del 9-------CTAGATATAAA -9

GGGAGACCCAAGCTGGCTAGCGCTC--------------del 38------------TAGATATGAAATC -38

CTAGAGCAACACGGAAGGA**ATTACCCT**--------del 9----------**A**TCTAGATATGAAA -9

CTAGAGCAACACGGAAGGA**ATTACCCTGTTA**------------del 131---CACACCTGTGC -131

CTAGAGCAACACGGAAGGA**AT**-------del 8--------------**TATCCCTA**TCTAGATATAAA -8

CTAGAGCAACACGGAAGGA**AT**-------del 8--------------**TATCCCTA**TCTAGATATAAA -8

AAAATGTCGTAACAACTCCG-------------del 217--------------ATTCCTTGCGGTCCGAAT -217

CTAGAGCAACACGGAAGGA**ATTACCCTGTTA**---------------del 209----------CACCAT -209

CTAGAGCAAC----------------------del 23------------------------**CCTA**TCTAGATATAAAAT -23

ACTATAGGGAGACCCAAGCT--------------------del 320-------GTCACTCAAGGGAAG -320

CTAGAGCAACACGGAAGGA**ATTACCC**--------del 8-----**CTA**TCTAGATATAAAAT -8

CTAGAGCAACACGGAAGGA**ATTACCCT**-------del 9--------**A**TCTAGATATGAAAT -9

CTAGAGCAACACGGA--------------del 222----------GACCACCATGTGCCGAGCCATC -222

CTAGCGCTCTAGAGCAA------------del 40---------------TCACGCCATGTAGTGTATT -40

CTAGAGCAACACGGAAGG**AATTACCCT**-----del 9-------**A**TCTAGATATAAAATCA -9

CTAGAGCAACACGGAAGG**AATTACCCT**------del 9------**A**TCTAGATATGAAATCA -9

- **Deletion / Insertion : 15/120 (12.5%)**

AGACCCAAGCTGGC----------del 59/ins 3--------------ACGCCATGTAGTGTATTGA -59+3

TGGTGATGCGGTTTTGGCA-------------------del 713/ins 171----------------------------- -713+171

GCAGTACATCAATGGGC-------del 269/ins 303----- ATTACCCTGTTATCCCTATC -269+303

CTAGAGCAACACGGAAGGA**ATTACCCT**----del 9/ins 8----**TA**TCTAGATATGAAA -9+8

CTAGAGCAACACGGAAGGA**ATTACCCTGT**-----del 80/ins 3------------CCATTCG -80+3

CTAGAGCAACACGGAA------------del 12/ins 4---**TTATCCCTA**TCTAGATATGAAA -12+4

TTTGGCAGTACATCAATGGGC------del 273/ins 311--------- AGGAATTACCCTGTT -273+311

CTAGAGCAACACGGAAGGA**A**------------del 14/ins4-------**CTA**TCTAGATATAAAAT -14+4

AGAGCAACACACGGAAGGA**ATTATCCC**----del 18/ins16-------------GATATAAAAT -18+16

CTCTAGAGCAACACGG---------del 190/ins 6----------------GGCTCAGATTCCCAAC -190+6

CTAGAGCAACACGGAAGGA**AT**---------------del 14/ins1----**TA**TCTAGATATAAAAT -14+1

ATAGGGAGACCCAAGCTGGCTAG--------del 202/ins10---------CACCTGTGCAAGA -202+10

CTAGAGCAACACGGAAGG---------del 21/ins3-------------TAGATATGAAATCACGC -21+3

CTAGCGCTCTAGA----------del 24/ins3---------------**TATCCCTA**TCTAGATATAAAA -24+3

CTGGCTAGCGCTCTAGAGCAAC-----del 9/ins 14--ATTACCCTGTTATCCCTATCT -9+14

**CtIP siRNA, Close DNA ends (CD4-34bp) GCK20 cells**

Total number of sequences :86

- **HiFi: 59/86 (69%)**

CTAGAGCAACACGGAAGGA**ATTACCCTGTTATCCCTA**TCTAGATATGAAA 51X

CTAGAGCAACACGGAAGGA**ATTACCCTGTT----CCCTA**TCTAGATATGAAA 1X

CTAGAGCAACACGGAAGGA**ATTACCCTGT------CCCTA**TCTAGATATGAAA 2X

CTAGAGCAACACGGAAGGA**ATTACCCTG--TATCCCTA**TCTAGATATGAAA 5X

- **Insertion : 4/86 (5%)**

CAACACGGAAGGA**ATTACCCTGTTAT**---ins 67-----**CCCTA**TCTAGATATGAAAT +67

CAACACGGAAGGA**ATTACCCTGTTATCCCTA**--------ins 17-----------TCTAGATAT +17

CAACACGGAAGGA**ATTACCCTGTTATCCCT**-----ins 7-----**A**TCTAGATATGAAAT +7

CAACACGGAAGGA-----ins 33--------**ATTACCCTGTTATCCCTA**TCTAGATATG +33

- **Deletion : 15/86 (17%)**

CTAGAGCAACACGGAAGGA**ATTACCCT**--------del 9-----**A**TCTAGATATGAAA -9

CTAGAGCAACACGGAAGGA**AT**-----del 8------**TATCCCTA**TCTAGATATGAAA -8

CTAGAGCAACACGGAAGGA**ATTAC**---del 5--**TATCCCTA**TCTAGATATGAAA -5

CTAGAGCAACACGGAAGGA**AT**-----del 8------**TATCCCTA**TCTAGATATGAAA -8

CTAGAGCAACACGGAAGGA**AT**-----del 8------**TATCCCTA**TCTAGATATGAAA -8

CTAGAGCAACACGGAAGGA**ATTACCCTGT**------del 9-------CTAGATATGAAA -9

CTAGAGCAACACGGAAGGA**ATTACCCTGTTATC**----del 6—TAGATATGAAA -6

CTATAGGGAGACCCAAGCT-----------del 68---------GTAGTGTATTGACCGATTCC -68

CTAGAGCAACACGGAAGGA**ATTACCCTGT-----**--del 9-------CTAGATATAAAA -9

CTAGAGCAACACGGAAGGA**AT**-------del 11---------**CCCTA**TCTAGATATAAAA -11

TAGCGCTCTAGAGCA---------------del 44-------CGCCATGTAGTGTATTGACCGA -44

CTAGAGCAACACGGAAGGA**ATTAC**-------------del 172------------TGGCCAGAGG -172

TTACTGGCTTATCGAA-----------del 92----------ATCACGCCATGTAGTGTATTGAC -92

CTAGAGCAACACGGAAGGA**ATTACCC**-----del 8-------**CTA**TCTAGATATAAAAT -8

CTAGAGCAACACGGAAGGA**ATTACC**-------del 10--------**TA**TCTAGATATAAAAT -10

- **Deletion / Insertion : 8/86 (9%)**

CACGGAAGGA--------------del 348/ins 1----------------TCCCAGAAGAAGATCACA -348+1

GACTTTCCAAAATGTCGTAACA----------del 183/ins 1--------------CTATCTAGAT -183+1

CTAGAGCAACACGGAAGGA------del 28/ins 4-----------------------AAATCACGCC -28+4

CTAGAGCAACACGGAAGGA------del 28/ins 4-----------------------AAATCACGCC -28+4

CTAGCGCTCTAGAGCAACACGGAAGG------del 238/ins 2---------ATCTCTCTTAG -238+2

CTAGCGCTCTAGAGCAACACGGAAGG------del 238/ins 2---------ATCTCTCTTAG -238+2

CTATAGGGAGACCCAAGC------------del 300/ins 337------------GCAGCTGTCACAA -300+337

AAATCAACGGGACTTTC--------------------del 613/ins 568--------------------------------- -613+568

**53BP1+CtIP siRNA, Close DNA ends (CD4-34bp) GCK20 cells**

Total number of sequences :78

- **HiFi: 45/78 (58%)**

CTAGAGCAACACGGAAGGA**ATTACCCTGTTATCCCTA**TCTAGATATGAAA 42X

CTAGAGCAACACGGAAGGA**ATTACCCTGT--ATCCCTA**TCTAGATATGAAA 3X

- **Insertion : 1/78 (1%)**

CAACACGGAAGGA**ATTACCCTGT**--ins 3--**TATCCCTA**TCTAGATATGAAATC +3

- **Deletion : 23/78 (29%)**

CAACACGGAAGGA**ATTACCCTGT**-------del 9-------CTAGATATGAAATCACGCC -9

CAACACGGAAGGA-----------------del 112---------------GGGGATCCTCTAGAGTCGA -112

CAACACGGAAGG-----------------del 87------------------TCACCCATTCGAATTCGAGC -87

CAACACGGAAGGA**ATTACCCT**----del 9----------ATCTAGATATAAAATCACGCC -9

CAACACGGAAGGA**ATTACCCTG**------del 4-----**CCCTA**TCTAGATATGAAATCA -4

CAACACGGAAGGA**AT**--------del 8------------**TATCCCTA**TCTAGATATGAAATCA -8

CAACACGGAAGGA**ATTACCCTGTTATC**------del 6----TAGATATGAAATCACG -6

CAACACGGAAGGA**ATTACCCT**------del 9-----------**A**TCTAGATATGAAATCACGC -9

CAACACGGAAGGA-------------del 112-----------GGGGATCCTCTAGAGTCGAGCCC -112

CAACACGGAAGGA**ATTACCCT**--------------del 9-------**A**TCTAGATATGAAATCAC -9

CAACACGGAAGGA**AT**-----del 8--------------**TATCCCTA**TCTAGATATGAAATCAC -8

CAACACGGAAGGA-----------------del 112----------GGGGATCCTCTAGAGTCGAGCC -112

CAACACGGAAGGA-----------------del 112----------GGGGATCCTCTAGAGTCGAGCC -112

CAACACGGAAGGA**ATTACCCT**------del 9---------------**A**TCTAGATATGAAATCAC -9

CAACACGGAAGGA**ATTACCCTGTTATC**-----del 6---------TAGATATGAAATCAC -6

CAACACGGAAGGA**ATTACCCTGTTAT**---------------del 222------GCCATCTCTCT -222

GCAACACGGAAGGA**AT**-------del 8-------------**TATCCCTA**TCTAGATATAAAATC -8

CAACACGGAAGGA**ATTACCCT**---------del 9---------------**A**TCTAGATATGAAATC -9

CAACACGGAAGGA**ATTACCCTGTTATCC**-----del 1----**TA**TCTAGATATAAAAT -1

CAACACGGAAGGA**ATTACCC**------------del 215--------------ACCATGTGCCGAGC -215

CAACACGGAAGGA**ATTACCC**------------del 215--------------ACCATGTGCCGAGC -215

CAACACGGAAGGA**ATTACCCT**----del 9------------------------**A**TCTAGATATGAAA -9

CAACACGGAAGGA**ATTACCCT**--------del 9--------------------**A**TCTAGATATAAAA -9

- **Deletion / Insertion : 9/78 (12%)**

CAACACGGAAGGA**ATTACCCTGT**---del 2/ins 2---**TCCCTA**TCTAGATATAAAAT -2+2

CAACACGGAAGGA**ATTACCCTGT**---del 2/ins 2---**TCCCTA**TCTAGATATAAAAT -2+2

TCCCAAGCTGGCTAGCGCTCTA-------------------del 438/ins 28-------------------------- -438+28

TGGTGATGcgGTTTTGGC------------------------------del 250/ins 237---------------------- -250+237

CAAGTCTCCACCCCATTGACGTC---------del 251 /ins 14------------AAATCACGCCA -251+14

GGAGGTCTATATAAGCAGAGCTCTCTG-----del 126/ins10-----AATCACGCCATGT -126+10

CAACACGGAAGGA**ATT**----------del 6/ins 1--------**TATCCCTA**TCTAGATATAAAA -6+1

CAACACGGAAGGA**ATTACCCTGTTAT**-----del 341/ins198------- CTGCGAGAGTTC -341+198

CAACACGGAAGGA**ATTACC**-------del 4/ins 4-------**TATCCCTA**TCTAGATATGAA -4+4
