# Supplementary material for: 53BP1 Protects against CtIP-Dependent Capture of Ectopic Chromosomal Sequences at the Junction of Distant Double-Strand Breaks
Source: PLoS Genet. 2016 Oct 31;12(10):e1006230. doi: 10.1371/journal.pgen.1006230 (PMC5087911; doi:10.1371/journal.pgen.1006230)
Supplement: S4 Supplementary information — (DOCX) [file pgen.1006230.s004.docx]

***S4***

**Insertions on distant DSEs (CD4-3200bp) GC92 cells**

Lowercase: surrounding vector sequence

Capital letters: insertions

Yellow boxes: micro-homologies ≥2bp

Grey boxes: error free copy of the original sequence, supposed SCE

**si Control:**

**(**12 insertions ≥ 45bp over 190 repair sequences total)

- CD4-3200bp reporter duplication: 4/12 (33%)

1: non-templated nucleotide; 2-66: CD4-3200bp reporter duplication (100% identity) ; 67-111: CD4-3200bp reporter duplication (100% identity) ; 112-148: non templated nucleotides

GGCCTCTGAGCTATTCCAGAAGTAGTGAGGAGGCTTTTTTGGAGGCCTAGGCTTTTGCAAAAAGCTAACTTGTTTAxTTGCAGCTTATAATGGTTACAAATAAAGCAATAGCGTGTTCGACTCTGTGCGACCCCAGGGACGGGGGATC

1-412: pCOH-CD4 duplication (100% identity); 413-427: non templated nucleotides

ccgccccaCGTTATCTGTTGTGGGATGAAGCCATATAGACAACGAAGGTGGGCTGGGGGAGTTTGGAGCTGGAGTTCTGGAAGAGCCAAGAGCATCCTTGCGAAACGGACCCCAACACTTCACATACCAGGTCCACCTTCTGACCAAGTTCGGCGTCCATTTTCTTTGGAAAGATTCGGAGTTCGGGTGCCTGTGGCTTAGCTTCTCCACTCCCCAGGATAATCGACTCACCCAGCAGCAGCAGGTTCAGCGACAGAAAGCGGGTCAACGGTGAGGCCATGGTGTGCTCTCCCCTAGAGCCCTACGGAATTCGAGCTCGCCCGGGGATCTCGAGGTCACCCTGACGGTGTCGTCCATCACAGTTTGCCAGTGATACACATGGGGATCAGCAATCGCGCATAATCGATATTACCCTGTTATATGGGAGTTTAGATA

1-3: non templated nucleotides 4-298: pCOH-CD4 duplication (79% identity); 299-300: non templated nucleotides

TTTCATCAATGGGCGTGGAGAGCGGTTTGCCTTACGGGGATCTGCGAGTCTCCAGTCTACTGACGTCAATCGGCATTTGTTATGGCTTCAAACTCCACAAGCCTCTCCAAAATCTAATAACACGTACCAACCACTGACGCAAATCGCAAGTAGGCGTGTGCGGTGTGAGGTCTATATAAGCAGAACTCTGAGCCCAACTAGAGACTCCAGAGCTCACTGCTTAATCGAATTTAATACGTCTCACTATAGGGAGTAGCAAGCTGCCTAGCGCTCTAGACCATCGCGGAACGAATTAGCCAG

1-8: non templated nucleotides 9-300: pCOH-CD4 duplication (70% identity); 301-306: non templated nucleotides

TTAGTGATTCAATGTGCGCAGATCACCGTTGGACTCACATAGATTTCCAAGTCTCCGCGACTTCCACGTCTCCAGGACTTTGTTTTCACACCGAATTCAACGTGACACTCCAATCAGTCGTACCATCTCAAATGCAGTGACACATCCGCCCCGTAGACGCATACGGTCGGAGGTCGAGATCAGCAGAACGTTCAGATAAACAGGAGACTCCGCTTAACTAGAGATTATAGAAATTAATGCGACTCAGTATAGGATGAGACAAGCTGTAGAGAGCTCCAAA CCGACTCGGAAGGAAGTACCACTCTG

- Chromosomal insertion: 8/12 (67%)

1-334: chromosome 8 CHM1_1.1 77332270 to 77332603

ttaccctgtAAATAACCTATTTTTTATTTATTTGATTTCTGTGAGTATCATTCTACCTCCGATCTTCATGATTCTTGACTCTGTAGAAAAAAAAAGATCTTGTCTTTAAGATAACTGTATTTTTACTTAATCAAAACAATAGCAATTTATAATTTATAAGGTATAGCATAGTGTTAAAAGAATTTTATCTCCTTGGCTCTTCTTAAATATCGTTTACAAATTCAAATATGCAAGTCCAAATATGGCCATTTATGTTGCCTCCCAAAACCCATGACATCTCTTCCCTCTCAGGGTTAATGGGTCAAAGATGATTCTGACGTGAATACATTGTGCTTTGTATAGTctttgat

1-172: chromosome 9 CHM1_1.1 102756217 to 102756388; 173-191: non templated nucleotides

tttggcaAGGTGGTGCTTTCCTGTGATCATGCTCAGTGCTGACAGGCAGGCTAAGGCTCGGATATCAAGGTTCAGGCTCTGCAAATTTCAGGAAAAGTCTTTAATAGAGTGGAACCACTCCACATTTGCCGGAATCCACCGAAGTCGATGCCTGACAAGTCCACTGCAGAACCCAAACTCCATCAAACCATATTCCCT

1-2: non-templated nucleotides; 3-240: Repeated sequence non Alu ; 241-258 : non-templated nucleotides

AAACTCTGTTTGTGAAGCCTGCCAGTGGATATTCGGACCTCTTTGAGGCCTTCGTTGGAAACGGGATTTCTTCATATTATGCTAGACAGAAGATTTCTCAGTAACTTCTTTGTGTTGTGTGTATGCAACTCACAGAGTTCAACCTTCCTTTAGACAGAGCAGATTTGAAACACTCTTTTTGTGGAATTTGCAAGTGGAGATTTCAAGCGCTTCGATGCCAATGGTAGAAAAGGAAATATCAGGTTATTGTCTCATGAG

1-227: *Alu sequence*; 1-467: chromosome 14 CHM1_1.1 75601794 to 75602260

*gttttggcTCACTACAACCTCCGCCTCCCGGGTTCAAGTGATTCTCCTGCCTCGGACTCCTGAGAGGCTGGGACTACAGGCACATGCCACGACACCCAGCTAATTTTTGTATTTTTAGTAGAGACAGGGTTTCACCATGTTGGCCAGACTGGTCTCGAATTCCTGACCTCAGGTGATCCGCCCGCCTCGGCCTCCCAAAATGCTGGAATTATAGGCGTCAGCCACTGCGCCCTGCTAATGGACAACCTTTAAACCTACATGTTTGGCTATTTCTTAGTATTTTAGGGTTGCTTCGTGCTCTGGCCTGGGTTTGCAGGAAAAATGGGAGAGAAGAGGAGAAAAGGGCTGGAAGAGGCTTGGCCATGGTAGAAGGCATGGGTGGGTGTGAGGAGAAGGAAACAGAGTGATGGGCCAGCAAGGAGTCACAGGGTTTCGGGATCTGGGCTAGCGACCCTCCTGGCCTCCAATGGGCTAAcagcatg*

1-149: chromosome 1 CHM1_1.1 211271134 to 211271282; 150-151: non-templated nucleotides

ttatccGTTTCCCTCCCCCAACGTTTTCTTTTACTTCTACTCACAGGAACTCCATATTTTCAGTGTTAAAGGCAGTCCAGGAAATAACCCTCTGTATTAGTTTACAGATAGCTGTTAATGAATTAACAAGTTACTTTCCTTGGGATATTTGCAATCC

1-85: chromosome 16, alternate assembly CHM1_1.1 3221396 to 3221480; 86-88 : non-templated nucleotides

ggttttggcCCAACAGGATTTGACCCTGAGGCCCACTCTCACCCTAATCATAACCGCAAAACCACCAGCGCCTGGAGAGAGAGTGAGAGAGAAATTG

1-160: Homo sapiens chromosome 16, alternate assembly CHM1_1.1 3221396 to 3221556; 161-164 : non-templated nucleotides

ggttttggcCCAACAGGATTTGACCCTGAGGCCCACTCTCACCCTAATCATAACCGCAAAACCACCAGCGCCTGGAGAGAGAGTGAGAGAGAAACAGAAACGGAGCGAGTGTGTGTCGCTGTGATGCCCTTTGCCGTCGCTGCTCATCCCCAGTGACCTCCTGAACTTTCAGG

1-153: Homo sapiens chromosome 16, alternate assembly CHM1_1.1 3221396 to 3221548; 154 : non-templated nucleotides

ggttttggcCCAACAGGATTTGACCCTGAGGCCCACTCTCACCCTAATCATAACCGCAAAACCACCAGCGCCTGGAGAGAGAGTGAGAGAGAAACAGAAACGGAGCGAGTGTGTGTCGCTGTGATGCCCTTTGCCGTCGCTGCTCATCCCCAGTGACCTCCTA

**si53BP1**

**(**24 insertions ≥ 45bp over 163 repair sequences total)

- CD4-3200bp reporter duplication : 10/24 (42%)

1-17: non templated nucleotides ; 18-59: pCOH-CD4 duplication (95% identity)

TTGACTCACGGNNTTNNTGATGNCGTTTTGGCAGTACATCAATGGGCGTGGATAGCGGT tcccta

1-61: pCOH-CD4 duplication (99% identity); 62-129: pCOH-CD4 duplication (100% identity); 130: non templated nucleotides

ccctgttatTTGTGAAATTTGTGATGCTATTGCTTTATTTGTAACCATTATAAGCTGCAATAAACAAGTT*AGCTTTTTGCAAAAGCCTAGGCCTCCAAAAAAGCCTCCTCACTACTTCTGGAATAGCTCAGAGGCCGA*T

1-61: pCOH-CD4 duplication (99% identity); 62-129: pCOH-CD4 duplication (100% identity); 130: non templated nucleotides

ccctgttatTTGTGAAATTTGTGATGCTATTGCTTTATTTGTAACCATTATAAGCTGCAATAAACAAGTT*AGCTTTTTGCAAAAGCCTAGGCCTCCAAAAAAGCCTCCTCACTACTTCTGGAATAGCTCAGAGGCCGA*T

1-2: non templated nucleotides ; 3-144: pCOH-CD4 duplication (100% identity)

ACCAACGGTGAGGCCATGGTGTGCTCTCCCCTAGAGCCCTACGGAATTCGAGCTCGCCCGGGGATCTCGAGGTCACCCTGACGGTGTCGTCCATCACAGTTTGCCAGTGATACACATGGGGATCAGCAATCGCGCATAATCGATattaccctgttatc

1-290: pCOH-CD4 duplication (100% identity)

gtcgtaacaCTTCACATACCAGGTCCACCTTCTGACCAAGTTCGGCGTCCATTTTCTTTGGAAAGATTCGGAGTTCGGGTGCCTGTGGCTTAGCTTCTCCACTCCCCAGGATAATCGACTCACCCAGCAGCAGCAGGTTCAGCGACAGAAAGCGGGTCAACGGTGAGGCCATGGTGTGCTCTCCCCTAGAGCCCTACGGAATTCGAGCTCGCCCGGGGATCTCGAGGTCACCCTGACGGTGTCGTCCATCACAGTTTGCCAGTGATACACATGGGGATCAGCAATCGCGCATAATCGATattaccctgttatc

1-3: non-templated nucleotides ; 4-141: pCOH-CD4 duplication (96% identity)

TGGGGTGATGGCCGTTTGGCAGTACATCAATGGGCGTGGATAGCGGTTTGACTCACGGGGATTTCCAAGTCTCCACCCCATTGACGTCAATGGGAATTTGTTTTGGCACCAAAATCAACGGGACTTTCCAAAATGTCGTAActatc

1: non-templated nucleotides ; 2-304: pCOH-CD4 duplication (98% identity)

TTGGTGATGACGTTTTGGCAGTACATCAATGGGCGTGGATAGCGGTTTGACTCACGGGGATTTCCAAGTCTCCACCCCATGGACGTCAATGGGAATTTGTTTTGGCACCAAAATCAACGGGACTTTCCAAAATGTCGTAACAACTCCGCCCCATTGACGCAAATGGGCGGTAGGCGTGTACGGTGGGAGGTCTATATAAGCAGAGCTCTCTGGCTAACTAGAGAACCCACTGCTTACTGGCTTATCGAAATTAATACGACTCACTATAGGGAGACCCAAGCTGGCTAGCGCTCTAGAGCACACGTAGTTCG

1: non-templated nucleotides ; 2-304: pCOH-CD4 duplication (98% identity); 305-311: non-templated nucleotides

TTGGTGATGACGTTTTGGCAGTACATCAATGGGCGTGGATAGCGGTTTGACTCACGGGGATTTCCAAGTCTCCACCCCATGGACGTCAATGGGAATTTGTTTTGGCACCAAAATCAACGGGACTTTCCAAAATGTCGTAACAACTCCGCCCCATTGACGCAAATGGGCGGTAGGCGTGTACGGTGGGAGGTCTATATAAGCAGAGCTCTCTGGCTAACTAGAGAACCCACTGCTTACTGGCTTATCGAAATTAATACGACTCACTATAGGGAGACCCAAGCTGGCTAGCGCTCTAGAGCACACGTAGTTCG

1-213 : pCOH-CD4 duplication (99% identity); 214: non-templated nucleotides

ggaaggCTAGCGTTTAAACTTAAGCTTGGTACCGAGCTCGGATCCGTCGGAACTCAGAAGTCGCTAATCGCCGACCAGTGCGATGGCACCCTGCACGCTGCTCCTGCTGTTGGCGGCCGCCCTGGCCCCCACTCAGACCCGCGCGGGCCCACATTCGCTGAGGTATTTCGTCACCGCCGTGTCCCGGCCCGGCCTCGGGGAGCCCCGGTTCATCGCTGTG

1-24: non-templated nucleotides; 25-308: pCOH-CD4 duplication (93% identity)

AATCAACGTTTGTGAAAGGCCGTTTGGCAGTACATCAATGGGCGTGGATAGCGGTTTGACTCACGGGGATTTCCAAGTCTCCACCCCATTGACGTCAATGGGAATTTGTTTTGGCACCAAAATCAACGGGACTTTCCAAAATGTCGTAACAACTCCGCCCCATTGACGCAAATGGGCGGTAGGCGTGTACGGTGGGAGGTCTATATAAGCAGAGCTCTCTGGCTAACTAGAGAACCCACTGCTTACTGGCTTATCGAAATTAATACGACTCACTATAGGGAGACCCAAGCTGGCTAGCGCTCTAGAGCaatca

- Chromosomal insertion: 12/24 (50%)

1-3: non-templated nucleotides ; *4-50 : Alu sequence* ; 5-321 : chromosome 22 CHM1_1.1 37899303 to 37899616 ; 322-328 : non-templated nucleotides

CTA*CCAAAGTGCTGGGATTACAGGCATGAGCCACCGCGCCCGGCCAGAAG*CTGATGTTTTGAACCACAGCCCCAGGGAGTTTCTTGGATCAGGCAAGTCTGGGAATCCTAGAGGCTGCGTATCAGGTCCTTCTTTGAAGACTCTTCTGGTGAAACACCTTCCGCCCACCTGTCAACATGTCCTTGGGGTAAAGCCAACTTTCTCTTCTTCTCAATTAATAATAATGTTTCCTAACTGGAATTGGGTGCACACTAGTGCCAGGTGCTTTCTAAAATATAAATTAATTAATTTCATCCACCAGTAGCCCTCTGGGGCAATATTAGTTAAC

1-34:non-templated nucleotides ; 35-143 : Chromosome 14 CHM1_1.1 89829383 to 89829491 ; 144-147 : non-templated nucleotides ; 148-283 : Chromosome 14 CHM1_1.1 46626273 to 46626408

CATACAATACATTTCATGCCATCTCTATCAAAATAACAGGCCAGGGGCAATGGCTCATGCCTGTAATCCAAGCACTTTGGGAGGCCAAAGTTGGTGGATCATGAGGTCAAGAGCTCAAGATCAGCCTGGCCAACTTGGTGAAAAGATACTAAATCTTTTTGAAACCTTGTGTATGTCAGGCTGTCTTTGCATTATAATTCCAGCAAGGTGATCTTGCTAAGAATTTGTTGCAAATAGCTTTGGTCCTTAATGTCACAACCTCCCTCCTGCGTGACTAAGATAGctcttaggcg

1-380: Chromosome 8 CHM1_1.1 1751679 to 1752058 ; 2-208 : Alu sequence

ttgg cTGCCTCCCAGGTTAAAGCGATTCTCCTGCCTCAGCCTCCCGAGTAGCTGGGACTACAAGCACGAGCCACCACGCCTGGTTAATTTTTTGTATTTTTAGTAGAGACGGGGTTTCACCGTGTTGGCCAGGATGATCTCGATCTCTTGACCTCATGATCTGCTGCCTTGGCCTCCCAAAGTGCTGGGATGACAGGTGTGAGCCATGGCGCCTGGTGTGATTTTTCTGTAACTACATCCTACTTATTCTGGACTAATGAGAAAATAGCAAGGGTGTCCATCTTCATCAACACTGCCTTGTTTGGACAGTTTGGTACAGGCTTCAAGGTTTCAGGCCTTAAGCTTTTGCAAATCACTTGCTCAGAAAAACTGNNGACAGCAGGCCcggca

1-49: non-templated nucleotides ; 50-73 : Chromosome 5 CHM1_1.1 58450091 to 58450114 and Chromosome 3 CHM1_1.1 52758451 to 52758473 ; 74-84 : non templated nucleotides

CATCTACCAACTCGCAGCCCCTGGCGCACTACCTTCTAGCGCTGGACGAACCCAAATCTCCTATCCAGATATGAAATCACGCCC

1-21: non templated nucleotides ; 22-132 : Chromosome 16 CHM1_1.1 3221393 to 3221504 ; 133-189 : non templated nucleotides

AGGCAGTTGGAGATGCGGGCAGGCCCAACAGGATTTGCAGTGTAAGGCTCTCTCTCAAGGATCAGGCTCGCAAAATCTCCAGCACCGTGAGTGAGAGAGAGGAAGCACCAGAAACGGACCGAGAGTGTGTCGACGTCATTGCCTGACAAGTAGAGCCCCAGCACAGATCACATTCAGACGTAAATAATT

1-21: non-templated nucleotides ; 22-137 : Chromosome 16 CHM1_1.1 3221393 to 3221508 ; 138-190 : non-templated nucleotides

AGGCGGTTGGTGATGAGGGCAGGCTCAGCAGGATTTGAACCTGTAAGGCTCTCTCTCAATAATCAGGCTCGGAAAATCTCCAGCACCGGGAGTGAGAGAGAGGAAGCACCAGAAACGGAGCGGGAGTGTGTCGCCGTCGTACCTGCCCGTAGATTTCATAGACCAGAACATCATCTGACCGTGGTAACTT

1-86: Chromosome 8 CHM1_1.1 77339526 to 77339611; 87-95: non-templated nucleotides

tctagagcaaTACTCCGGATTAATAGTCTCAGTTTTCCAGGAAAGCATGAAGAATGTGGCTCACGCATGGTAGAGAGTTAGAACTATTACTTCATTTCCGGAGCA

1-110: Chromosome 16 CHM1_1.1 3221396 to 3221505; 111-166: non-templated nucleotides

cggttttggcCCAACAGGATTTGACCCTGTGGTCCACTCTCGCAATAATCATAATCGGAAAATCTCCAGCACCGGGAGAGAGAGAGAGGGAGAACCAGAAACGGAGCGAGTTTGTGTCGCCCTCAGGCCTGCCCGTAGACTGCATAGCACAGAACTCCTGCAGTGTTAATCCGCCC

1-14: non templated nucleotides; 15-246: Repeated sequence non Alu; 247-308 : non-templated nucleotides

TTCTATCAAAGATTATTTTCTTTgTAAATTACCCAgTCTCTGGTATTTCTTTATAGCAACATGAGAAGGAACTAATACAGAAAATTCATACTGAGGAGTGGAGCATTGCCATAAAAATACCTGAAAATGAAGCAAGTTTGCAACTGGGTAATGGGTAGAAGTTGGAAAATTGTGAAGAGTTCAGAAGAAGACAAGGAGATGAGGGAAAGTTTGGAACTTCTTAGAGACTTGTTGAATGGTTGTGACTTTATAGTCTCAGAGTCCTTTATAGCAGTGTGAGAACAAACTAATACAAATGTGTTTTCATG

1-5: non-templated nucleotides: 6-193 : Alu sequence ; 194-204: non-templated nucleotides

TCAGGGCAACCTCTGCCTTTCGGGTTCAAGTGATTCTCCTGCCTCAGCCTCCCAAGTAGCTGAGACTACAGGCACGTGCCACCATGCCCGGCTAATTTTTGTATTTTTAGTAGAGGCGGGGTTTTGCCACGTTGGCCAGGCTGGTCTCAAACTCCTCACCTCAGGCGATCCACCTNCCTCANCCTCCCAAAGTCCNNGATACAG

1-26: Chromosome 8, alternate assembly CHM1_1.1, whole genome shotgun sequence 14587749 to 14587774. 27-69: non-templated nucleotides. 70-95: Chromosome X, alternate assembly CHM1_1.1, whole genome shotgun sequence 57347963 to 57347988. 96-123: non-templated nucleotides. 124-144 : Chromosome 12, alternate assembly CHM1_1.1, whole genome shotgun sequence 22620438 to 22620458. 145-175: non-templated nucleotides. 176-199 : Chromosome 10, alternate assembly CHM1_1.1, whole genome shotgun sequence 122755439 to 122755462

tggtTTTTTTTTTTTGTTTTGGCTGGTCCGCCAATCCACAGGGACGGGCGGACAGGGCTCACTGGAAAGGCCTTGTCTTCCCTCCATCCCCCGTCAATGAGAAGGGGGTTTGTTTAGTAAATCACCGAAACCATCCAAAATGTAATAACATTTCCACTCCATTGACGCCAATGAACCATAGGGGTGTAAGGTGGGAGGGGTAGGTGATCAGATCTATCGGTCTATCTAGGGAAAGCGAAGCCTCCAGGGATTTCGGGATTGATAAAACTCATAATACGGAGAAACGGGAGGCCCAAAGCTCTAGAGCCTCGGGGAAAAAGTGAACT

1-222: Homo sapiens chromosome 8, alternate assembly CHM1_1.1 94640757 to 94640978 ; 223-278: non-templated nucleotides

ggaaggGAAGAGGGAAAGGACATGGAATGTTTGAGAAGCAAACACACACTTCTCCCCAGAGTGATTCTGTGCATTAACCAGACACGTCTCCATTCTGCTCCCATGGACCTACTGCTGGTGATTCCCATTTGCAGTCGCCTAACCTACTACCCTTACGCTGTTCTCTGCTGTGTTCCTCAACCATCAGCATTTGGGGCAATTCTTCACAGCACAGGACTGCCCTCATATTTTAAATAAGAAATGTGTGATAACATCAAAAGAGATCACAGTCTCACCGTAAGGTC

- Other insertions: 2/24 (8%)

1-135: E. Coli genomic sequence

ccctACTCCTACGCGCGCAATTAACGAATCCACCATCGGGGCAGCTGGTGTCGATAACGAAGTATCTTCAACCGGTTGAGTATTGAGCGTATGTTTTGGAATAACAGGCGCACGCTTCATTATCTAATCTCCCAGCGTGgtcaca

1-78: E. Coli genomic sequence

gtacggCGCATCCTGACGGAACTCTGGCGTACGGCCCATGACTGCCGCCAGATCAAAGCGGAAACCATCGACGTGGCATCGACGtgggag

**siCtIP**

**(**5 insertions ≥ 45bp over 165 repair sequences total)

- CD4-3200bp reporter duplication: 4/5(80%)

1-29: pCOH-CD4 duplication (100% identity) ; 24-45: pCOH-CD4 duplication (100% identity)

GCTCGCCCGGGGATCCTCTAGAGTCGAGCTCGCCCGGGGATCCTC

1-79: pCOH-CD4 duplication (100% identity)

TGTTCCTGGCTTGCGTGCTGGGTGGCTCCTTCGGCTTTCTGGGTTTCCTTGGGCTCTGCATCCTCTGCTGTGTCAGGTG

1-149 pCOH-CD4 duplication (99% identity); 150-181 : non-templated nucleotides

ccaagctgTGGGCCCGCGCGGGTCTGAGTGGGGGCCAGGGCGGCCGCCAACAGCAGGAGCAGCGTGCAGGGTGCCATCGCACTGGTCGGCGATTAGCGACTTCTGAGTTCCGACGGATCCGAGCTCGGTACCAAGCTTAAGTTTAAACGCTAGCTAGCGCTCTAGAGCTCACAGTCTCACCGTAGTCCC

1-9 : non-templated nucleotides ; 10-147: pCOH-CD4 duplication (100% identity).

ATTACCCGTGGTGAGGCCATGGTGTGCTCTCCCCTAGAGCCCTACGGAATTCGAGCTCGCCCGGGGATCTCGAGGTCACCCTGACGGTGTCGTCCATCACAGTTTGCCAGTGATACACATGGGGATCAGCAATCGCGCATAATCGATattaccctgttatc

- Chromosomal insertion: 1/5 (20%)

1-139 : Homo sapiens chromosome 5, alternate assembly CHM1_1.1, whole genome shotgun sequence 57571114 to 57571252

ttaccctCATACTCCTAAGTTAAGTTTTCAATCTTGTCTGCTTATTAAGTTAGGTATGGTTCTTCCATAGGACTCAAATATAGAAGTACAGAGTCCTTTTCAGGCCATATTTAGTTCACTTTAACACTGTAAATAATGAACTCCGAttatcc

**si53BP1+siCtIP**

**(**3 insertions ≥ 45bp over 95 repair sequences total)

- CD4-3200bp reporter duplication: 1/3 (33%)

1: non-templated nucleotide ; 2-312: pCOH-CD4 duplication (99% identity); 312-323: non template nucleotides

TTGGTGATGAGTTTTGGCAGTACATCAATGGGCGTGGATAGCGGTTTGACTCACGGGGATTTCCAAGTCTCCACCCCATTGACGTCAATGGGAATTTGTTTTGGCACCAAAATCAACGGGACTTTCCAAAATGTCGTAACAACTCCGCCCCATTGACGCAAATGGGCGGTAGGCGTGTACGGTGGGAGGTCTATATAAGCAGAGCTCTCTGGCTAACTAGAGAACCCACTGCTTACTGGCTTATCGAAATTAATACGACTCACTATAGGGAGACCCAAGCTGGCTAGCGCTCTAGAGCAACACGGAAGGAATAGCCGTAGTTC

- Other insertions: 2/3 (67%)

1-74: I-SceI expression vector

agcgctcAAGTCAAGGCTTTTCTATGGAATAAGGAATGGACAGCAGGGGGCTGTTTCATATACTGATGACCTCTTTATAGCttccttgcg

1-76: I-SceI expression vector ; 77-83: non-templated nucleotides

agcgctcAAGTCAAGGCTTTTCTATGGAATAAGGAATGGACAGCAGGGGGCTGTTTCATATACTGATGACCTCTTTATAGCCAACCTTTG

**Insertions on distant DSEs (CD4-3200bp) GC49 cells**

Lowercase: surrounding vector sequence

Capital letters: insertions

Yellow boxes: micro-homologies

**si Control:**

**(**2 insertions ≥ 45bp over 80 repair sequences total)

- CD4-3200bp reporter duplication: 1/2 (50%)

1-220: pCOH-CD4 duplication (100% identity)

ttaccctTCTAGATAGGGATAACAGGGTAATATCGATTATGCGCGATTGCTGATCCCCATGTGTATCACTGGCAAACTGTGATGGACGACACCGTCAGGGTGACCTCGAGATCCCCGGGCGAGCTCGAATTCCGTAGGGCTCTAGGGGAGAGCACACCATGGCCTCACCGTTGACCCGCTTTCTGTCGCTGAACCTGCTGCTGCTGGGTGAGTCGATTATCCTGGGGatcccta

- Chromosomal insertion: 1/2 (50%)

1-118 Homo sapiens chromosome 11, alternate assembly CHM1_1.1 63893987 to 63894104

ctgttatACACAGCCAAACTTGGAGGAGGCATGTATTGTAATGAATGGATACGGTGAAGACCAATGAAAAGCTGAAGCAGCTTTCACCCTTCCCAGTCACACACCCCAAACTCGCTCTGCACCCTccctatctag

**si53BP1**

**(**6 insertions ≥ 45bp over 114 repair sequences total)

- CD4-3200bp reporter duplication : 6/6 (100%)

1-2: non-templated nucleotides ; 3-448 : pCOH-CD4 duplication (100% identity)

TGCCCTCATGGCAGAAAACAGTTTCGACGAATTCAGCTTCTCGTCCCACGTTATCTTGTTGTGGGATGAAGCCATATAGACAACGAAGGTGGGCTGGGGGAGTTTGGAGCTGGAGTTCTGGAAGAGCCAAGAGCATCCTTGCGAAACGGACCCCAACACTTCACATACCAGGTCCACCTTCTGACCAAGTTCGGCGTCCATTTTCTTTGGAAAGATTCGGAGTTCGGGTGCCTGTGGCTTAGCTTCTCCACTCCCCAGGATAATCGACTCACCCAGCAGCAGCAGGTTCAGCGACAGAAAGCGGGTCAACGGTGAGGCCATGGTGTGCTCTCCCCTAGAGCCCTACGGAATTCGAGCTCGCCCGGGGATCTCGAGGTCACCCTGACGGTGTCGTCCATCACAGTTTGCCAGTGATACACATGGGGATCAGCAATCGCGCATAATCGATattaccctgttatc

1-9: non-templated nucleotides ; 10-150 : pCOH-CD4 duplication (89% identity) ; 151-269: non-templated nucleotides

GTGGTGGGGCTCTCGGGGTTTTCCAAGTTTCCACCCCATTGAGGTCAAGGGGAGTTTGTTTGGCACCAAAATCATCGGGAATTTCCAAAAAGTTGTAACAAATCGGCCCCATTGAGGCAAATGGGCGTTAGGCGGGTACGGTGGGAGATCAAATTATGAGGCTTATTTGGCAATACAGCGATGCCCGTGGTTAGCGGTTTGACTAAAGTCGTTTTACTCATATTCAGGCGACCCAAGTCAATAGGACTTCATGGACGTCAACAAAGTAA

1-23 pCOH-CD4 duplication (100% identity) ; 25-36 pCOH-CD4 duplication (100% identity) ; 35-45 pCOH-CD4 duplication (100% identity)

gcaaCACGGAAGGAATTACCCTGTTATGTTTCATATCTAGGAATTACCCtgttatccc

1-219 : pCOH-CD4 duplication (99% identity) ; 220: non-templated nucleotides

attaccctgtCCCTAGCTAGCGTTTAAACTTAAGCTTGGTACCGAGCTCGGATCCGTCGGAACTCAGAAGTCGCTAATCGCCGACCAGTGCGATGGCACCCTGCACGCTGCTCCTGCTGTTGGCGGCCGCCCTGGCCCCCACTCAGACCCGCGCGGGCCCACATTCGCTGAGGTATTTCGTCACCGCCGTGTCCCGGCCCGGCCTCGGGGAGCCCCGGTTCATCGCGGTT

1-112 : pCOH-CD4 duplication (98% identity)

taccctAGCTAGCGTTTAAACTTAAGCTTGGTACCGAGCTCGGATCCGTCGGAACTCAGAAGTCGCTAATCGCCGACCAGTGCGATGGCACCCTGCACGCTGCTCCTGCTGTTGGCGGcccta

1-68 : pCOH-CD4 duplication (100% identity)

attacCCTGACGGTGTCGTCCATCACAGTTTGCCAGTGATACACATGGGGATCAGCAATCGCGCATAATCGATcctgtta
