# Supplementary material for: 53BP1 Protects against CtIP-Dependent Capture of Ectopic Chromosomal Sequences at the Junction of Distant Double-Strand Breaks
Source: PLoS Genet. 2016 Oct 31;12(10):e1006230. doi: 10.1371/journal.pgen.1006230 (PMC5087911; doi:10.1371/journal.pgen.1006230)
Supplement: S5 Supplementary information — (DOCX) [file pgen.1006230.s005.docx]

***S5***

**Insertions on close ends (34bp) GCK20 cells**

|  |  |  | **Origin of insertions**≥**45bp in the repair of CLOSE DSEs** | | |  |
| --- | --- | --- | --- | --- | --- | --- |
| siRNA | insertions ≥45bp | % of insertions ≥45bp coupled to a deletion >100bp | **Duplication of the EJ reporter** | **Ectopic chromosomal sequences(ECS)** | **Other** | Insertions ≥45bp borded by non-identified nucleotides |
| siControl | 1.5%  (2/135) | 0%  (0/135) | 0.7%  (1/135) | 0.7%  (1/135) | - | 100%  (2/2) |
| si53BP1 | 2.5%  (3/120) | 2.5%  (3/120) | 1.7%  (2/120 | 0.8%  (1/120) | - | 100% (3/3) |
| siCtIP | 2.2%  (2/86) | 1.1 %  (1/86) | 1.1%  (1/86 | 1.1%  (1/86) | - | 50%  (1/2) |
| si53BP1+siCtIP | 2.6%  (2/78) | 2.6%  (2/78) | - | 1.3%  (1/78) | 1.3% *  (1/78) | 100%  (2/2) |

*:Bacterial DNA

**siControl :**

**(2** insertions ≥ 45bp over 135 repair sequences total)

- CD4-3200bp reporter duplication: 1/2 (50%)

1-15: non-templated nuleotides ; 16-320: CD4-3200bp reporter duplication (100% identity); 317-360: CD4-3200bp reporter duplication (98% identity)

ATTTTGTGGGATGGGTTGGCAGTACATCAATGGGCGTGGATAGCGGTTTGACTCACGGGGATTTCCAAGTCTCCACCCCATTGACGTCAATGGGAGTTTGTTTTGGCACCAAAATCAACGGGACTTTCCAAAATGTCGTAACAACTCCGCCCCATTGACGCAAATGGGCGGTAGGCGTGTACGGTGGGAGGTCTATATAAGCAGAGCTCTCTGGCTAACTAGAGAACCCACTGCTTACTGGCTTATCGAAATTAATACGACTCACTATAGGGAGACCCAAGCTGGCTAGCGCTCTAGAGCAACACGGAAGGAATTACCCTATCTAGATATAAAATCACGCCATGTAGTGTATTGACCGATatatacacac

- Chromosomal insertion: 1/2 (50%)

*1-107: Alu sequence*; 1-115 *:* Chromosome 1 CHM1.1_1 26888777 to 26888891 ; 116-117 : non-templated nuleotides :

*GTAGAGACGGGGTTTCACCATGTTGGCGAGGCTGGTCTTGAACTCCCAACCTCAAGTGATCTGTCTGCCTCAGCCTCCCAAAGTGCTGAGATTCCAGGCATGAGCCA*TCACTCCTTA

**si53BP1:**

**(**3 insertions ≥ 45bp over 120 repair sequences total)

- CD4-3200bp reporter duplication: 2/3 (66%)

1-41: non-templated nucleotides; 42-213: CD4-3200bp reporter duplication (70% identity); *162-276: CD4-3200bp reporter duplication (75% identity)* ; 277-303 : non-templated nucleotides :

TGGTGAGCGCGTTTGTGTCAGGGCGTCAATCAGGTCTGCTCGCCGTTTGACTCACGGGGAGTTTCATGTGTCCACAACATCGACGTCAATGTGCGTAAGGTCGTGCCACATCATCAACATGGCTGTACATAATCTGGTGGCAACTCCGGCCCGATGTCGCAA*TAAGCAGATCTCTGTGTACAATTAGAGATCCAAATAAGCAGAGCTCTATGGAAATTAATAGGACTCACTATAGGGAGACTCAAGGAAACTAGTGCTCCAGAGCATCGCGGGAGG*CAATCTGCTGATCTCTCTAGACCACCC

1-33: non-templated nucleotides; 34-239: CD4-3200bp reporter duplication (65% identity); 240-311; non-templated nucleotides:

AGAGCGGGTTGTGTCACTACGTCATTCGGGTCTGCTCGCCATTGACGTCACGGGGAGTTTCATGTGTCCACACCATCGACGTCAATGTGAGAAAGGTCGTGCCACATACTCAACATGGACGCACATAATCTGGAGGCAACTCCGGTGCGAGGTCGCAATTAGCCGATATGTCTGTACGGTTAGAGGTCTCTATAAGCAGAGCTTTATGGAAATTTATAGGACTCACTATAGGGAGGCTCATGGAAATTAATGCTACTCAGCATCGCGGGAGGCAATCTGGTGATCTCTCTAGACCACCACGGCAGGG

- Chromosomal insertion: 1/3 (33%)

1-155: Chromosome 1 CHM1_1.1 693075 to 693229 ;156-171 : non-templated nucleotides :

tgatgttttggcaCGTTGCCAGTCCACCTGTCTCAGCCCCGCAAAGTGCTGGTATTACAGGAGTGAGCCACTGCACCCAGCATTTGCCAAGACCTTTGATGGCAGGCTTTTTCCAGGTGATCAGTCCTTGTCTGGTCTGGCTCTGCCCCACTCTCCTTCTCACCTAGTCGATCAAACAGCCTTG

**siCtIP:**

**(**2 insertions ≥ 45bp over 86 repair sequences total)

- CD4-3200bp reporter duplication: 1/2 (50%)

1-20: : non-templated nucleotides ; 21-335: CD4-3200bp reporter duplication (100% identity);

TGGCTGAGTCGCTTGCCCGATTGGCAGTACATCAATGGGCGTGGAAAGCGGTTTGACTCACGGCGATTTCCAAGTCTCCACCCCATTGACGTCAATGGGAGTTTGCTTTGGCACCAAAATCAACGGGACTTTCCAAAAAGTGGTAACAACTCCGCCCCACTGACGCAAATGGGCTGTAGGCGTGTTCGGTGGGAGGTCTATATAAGCAGAGCTCTCTGGCTAACTAGAGAACCCAGTGCTTACTGGCTTATCGAAATTAATACGACTCACCATAGGGAGACCCAAGCTGGCTAGCGCTCTAGAGCAACACGGAAGGAATTACCCTGTTATCCCTAACC

- Chromosomal insertion : 1/2 (50%)

1-67 : Alu sequence :

GGCTCACACCTGTAATCCCAGCACTTTGGGAGGCCAAGGCGGGTGGATCACCTGAGGTCAGGAGTTC

**si53BP1+siCtIP:**

**(**2 insertions ≥ 45bp over 78 repair sequences total)

- Other insertions: 1/2 (50%)

1-192: Bacterial DNA ; 193-206 : non-templated nucleotides

accctgttatGCGCGAATGGCTGCCAGTCCTTGTTCGCGCAAAAGGGCGGCGCAGGCCGCGTAGCCGAAGTGGTTTTCATGCGACATGTAGCTGGCTTCTCGATCGCTAAATATATCCTGGCTCCAGTAGTGTTCGAGTTTTCTCACCGCTGTGGGGTCATTAGCAACAACTTTTAACCACTCCTTAGAATGAAAATGAGTTATCCCTAACCGCCG

- Chromosomal insertion : 1/2 (50%)

1-227: Alu sequence; 228-236: non-templated nucleotides

gcggttttggcTCACTGCAACCTCCACCTCCTGGGGTCAAGCCATTCTCCTGCCTCAGCCTCCCGAGTAGCTGGGATTACAGGCACCCACCACCATGCCTGGCTAATTTTTGTATTTTAGTAGAGAAAGGGTTTCACCATGTTGGCTAGGCTGGTCTCGAACCCCTGACCTCAGGTGATCCGCCTGCCTTGGGCTCCCAAAGTGCTGGGATTACAGGCGTGAGCCACTGTGCCTGGCCTGTCTGCCA
